# Barriers and facilitators for the management of vertigo: a qualitative study with primary care providers

**DOI:** 10.1186/s13012-018-0716-y

**Published:** 2018-02-08

**Authors:** Anna-Janina Stephan, Eva Kovacs, Amanda Phillips, Jörg Schelling, Susanne Marlene Ulrich, Eva Grill

**Affiliations:** 1 Institute for Medical Information Processing, Biometry and Epidemiology, Ludwig-Maximilians-Universität München, Marchioninistraße 17, 81377 Munich, Germany; 2 German Centre for Vertigo and Balance Disorders, University Hospital, Ludwig-Maximilians-Universität München, Munich, Germany; 3 Institute for General Practice and Family Medicine, University Hospital, Ludwig-Maximilians-Universität München, Munich, Germany; 4 Munich Centre of Health Sciences, Ludwig-Maximilians-Universität München, Munich, Germany

**Keywords:** Implementation research, Physician behaviour, Primary care, Qualitative research, Guidelines

## Abstract

**Background:**

Although the management of patients presenting with vertigo and dizziness in primary care has been reported to be inefficient, little is known about the primary care providers’ (PCPs) perspectives, needs, and attitudes regarding vertigo management.

The objective of this study was to understand which challenges and barriers PCPs see when diagnosing and treating patients presenting with vertigo or dizziness. Specifically, we wanted to identify facilitators and barriers of successful guideline implementation in order to inform the development of targeted interventions.

**Methods:**

A theory-based interview structure was developed based on the implementation theory of capability, opportunity, and motivation for behaviour change (COM-B) using questions based on constructs from the Theoretical Domains Framework (TDF) and the Consolidated Framework for Implementation Research (CFIR). Transcripts of the semi-structured interviews were analysed using directed content analysis. The pathways through which guideline characteristics and supportive interventions affect the relationship between the PCPs’ perceived capability, opportunity, and motivation as well as their practice of managing vertigo patients were graphically presented using the COM-B model structure.

**Results:**

Twelve PCPs from Bavaria in Southern Germany participated in semi-structured interviews. Diagnostics posed the biggest challenge in vertigo management to the PCPs. Requirements for an acceptable guideline were stakeholder involvement in the development process, clarity of presentation, and high applicability. Guideline implementation might be effectively supported through educational meetings and sustained by organisational interventions.

**Conclusions:**

From the PCPs’ perspective, both guideline characteristics and interventions supporting guideline implementation may help resolve challenges in vertigo management in primary care. These results should be used to guide future interventions in the primary care setting to ensure successful and targeted patient management.

**Electronic supplementary material:**

The online version of this article (10.1186/s13012-018-0716-y) contains supplementary material, which is available to authorized users.

## Background

Vertigo and dizziness are symptoms which are encountered frequently in primary care. The aetiology of vertigo and dizziness is often multifactorial. Peripheral and central vestibular diseases are the most obvious and frequent causes; however, vertigo and dizziness can also be provoked by cardiovascular diseases, by polyneuropathy, or by medication, or they can have a psychosomatic origin [1]. The appropriate choice of treatment, e.g. liberatory maneuovers of misplaced otoliths, physiotherapy, prescription of medications, adjustment of medication regimes, or cognitive behavioural therapy, depends largely on the correct identification of the underlying condition [2]. Although, once correctly diagnosed, treatment of vertigo is mostly quite straightforward, considerable uncertainty about the management of patients presenting with vertigo remains in primary care [2, 3]. There is evidence that an accurate and specific diagnosis could have been assigned to up to 86% of vertiginous patients who had previously received a diagnosis of ‘unspecific dizziness’ by their primary and secondary care physicians [1]. The primary care provider (PCP) decides about the patient’s diagnostic path through the health care system, e.g. to initiate diagnostic procedures and treatment or to refer the patient o the appropriate specialist [4]. Without a targeted and correct first PCP assessment, patients are being sent off in the wrong direction from the very beginning of the diagnostic process, which may result in redundant or unnecessary procedures and medication intake. Vertigo and dizziness play a predominant role among health conditions that are frequently underdiagnosed [1]. There is sound evidence that diagnostic and therapeutic needs of patients with vestibular disease are often unmet [2], leading to chronification and the development of secondary, functional symptoms [5]. As 45% of vertigo and dizziness patients’ first contacts with the health care system occur on the primary care level [6], the PCPs’ expertise is of utmost importance.

In Germany and internationally, several specialist-driven evidence-based guidelines have been published [7–11], but only recently, the German College of General Practitioners and Family Physicians introduced the first German guideline for the management of vertigo specifically targeting primary care [12]. This presents a unique chance for action, since appropriate support measures accompanying guideline introduction have the potential to increase guideline use and effectiveness [13], for example through identifying and addressing barriers of adequate disease management. Nevertheless, little is known about the PCPs’ perspectives, needs, and attitudes specifically regarding vertigo management and the support they would need for successful vertigo guideline implementation.

### Objective

The objective of this study was to understand which challenges and barriers PCPs see when diagnosing and treating patients presenting with vertigo or dizziness and which facilitators and barriers may arise with respect to vertigo guideline introduction. The results of this study will inform the development of interventions to improve vertigo management in primary care.

## Methods

### Study design

We conducted qualitative semi-structured interviews with primary care providers who reported having patients with vertigo and dizziness among their clientele.

### Interview structure

Basing intervention development on theory allows the researcher to understand which critical points an intervention needs to address and, after implementation, to identify why or why not an intervention worked in a specific context or setting [14]. We decided to organise both the interview structure and the coding frame for content analysis alongside the implementation theory [15] of capability, opportunity, and motivation for behaviour change (COM-B) [16]. We chose this implementation theory because on the one hand, it summarises the pre-requisites of enacting a certain behaviour: capability, opportunity, and motivation. Capability (‘C’) describes the psychological (intellectual) ability and the physical (practical) skills to enact a certain behaviour. Opportunity (‘O’) is defined as the perceived influences of the social and physical environment which may enable or hinder a certain behaviour. Motivation (‘M’) describes the processes which activate or inhibit a certain behaviour. On the other hand, the COM-B theory also suggests specific intervention strategies to enhance each of these pre-requisites. For example, if missing capabilities were found to be a major barrier for vertigo guideline implementation, COM-B would suggest an intervention mainly focussing on training or enablement, which properly addresses these barriers [16].

Since the COM-B does not provide specific constructs which could be readily operationalized into interview questions, we used constructs from the determinant framework [15] Theoretical Domains Framework (TDF) [17]. TDF is one of the two most frequently used theories for the subject of guideline implementation [18]. We chose it because our target intervention will be focussing on behaviour change. The TDF is a summary of constructs of behavioural change theories which offers the possibility to be further combined and extended with other frameworks [19], e.g. with the Consolidated Framework for Implementation Research (CFIR) [20]. Because behaviour does not only depend on the individual but is also influenced by the context, such as the setting and the characteristics of the intervention that one tries to implement [21], we followed previous studies [22, 23] and completed the list of relevant constructs from the TDF with constructs from the CFIR regarding intervention characteristics, characteristics of the implementation process, and characteristics of the environmental context. With regard to the characteristics of individuals, TDF and CFIR partly overlap. For example, TDF lists ‘beliefs about capabilities’ and CFIR lists ‘self-efficacy’. In these cases, we kept only one of the two constructs in our list. In the next step, for each construct, an interview question was formulated, e.g. ‘How easy or difficult do you find it to manage vertigo patients? Why?’. Last, all questions were reviewed for their relevance with regard to our research objectives, and the interview structure was shortened considerably in order to keep the interviews within a maximum length of 30 min.

Following an introductory section and some warm-up questions, the first relevant question covered the perceived challenges in vertigo management (the behaviour ‘B’, in the COM-B model). Next, the facilitators and barriers of successful vertigo management were addressed with one main question for each of the three dimensions thought to influence this behaviour (capability, opportunity, and motivation).

Additional questions on attitudes and expectations regarding practice guidelines and potential accompanying implementation methods covered the secondary objectives of the study. If necessary, prompts were given based on the Cochrane Effective Practice and Organisation of Care Group (EPOC) [24] taxonomy of interventions, a comprehensive list of potential intervention methods in the health care setting.

The complete interview structure including auxiliary questions, the EPOC list of interventions, and instructions for the interviewer can be found in Additional file 1 (original German questions and English translation).

### PCP and practice characteristics

The participating PCPs’ socio-demographic characteristics and working environments can be expected to shape the experiences and attitudes they reported in the interviews. To gain a better understanding about the working context of our specific sample and to allow for potential comparisons with the samples of other interview studies, PCP and practice sociodemographic characteristics were collected using the paper-based Questionnaire of Chronic Illness Care in Primary Care (QCPC) [25]. Interview participants were characterised by sex, mean age and age range, professional experience, and catchment area of the practice.

The following practice characteristics were used to describe the patient population of participating PCP practices: estimated mean and range of percentage of patients aged 60 years and older, patients with at least two chronic diseases, patients with mental diseases, and patients with migration background. Practice size was characterised by mean number and range of employees in the practice and their professions as well as number of patients treated per week.

In addition, it was asked how frequently evidence-based guidelines were used for patient treatment.

Furthermore, we asked for participation frequency in trainings and quality circles and for individual selection criteria for trainings.

### Participant recruitment

Recruitment was based on an available list of 714 primary care providers from Munich and surrounding counties in Bavaria in Southern Germany. Of these, 77 PCPs had consented to recruit vertigo patients into an ongoing observational cohort study for our working group. As we realised in the course of this cohort study that vertigo patients are not necessarily evenly distributed over PCP practices, we decided to contact only those PCPs from the database who had recently included vertigo patients in the cohort (within the 6 months prior to February 1, 2016). These PCPs were invited via postal mail to participate in a 30-min semi-structured interview. Sample size was determined according to the principle of saturation: As long as new themes emerged, we continued including further PCPs in the study.

### Data collection and interview situation

Interviews were conducted either as face-to-face interviews in the PCP practice or as telephone interviews depending on both the physical distance of the PCP practice from the study centre in Munich and the PCPs’ preferences. In all interview situations, only the PCP and the interviewer(s) were present in a quiet and uninterrupted environment. Interviews were conducted by two female project scientists experienced in qualitative data collection (AS, MPH, PhD candidate and EK, PhD, MD).

All participating PCPs provided verbal consent to the audio-recording of the interview. No additional field notes were taken, and no repeat interviews were carried out.

### Pilot testing

The interview concept was tested on the first participating PCP who, after the interview, was asked for feedback on the interview situation as well as the structure and phrasing of questions.

### Data preparation

All interviews were audio-recorded and transcribed verbatim by MP, EK, and SU using free versions of ExpressScribe [26] or F4 [27] software. Each PCP was assigned a participant ID to allow for anonymization of the transcripts. AS performed the quality control comparing the original records and the transcriptions. Transcription was done clean verbatim and not logical; therefore, transcripts were not sent back to the interviewees for comments or correction. Instead, participants were offered the possibility to participate in a subsequent interdisciplinary expert workshop where the anonymised analysis results were presented and the PCPs were given the opportunity to comment on them and discuss the conclusions drawn by the research team.

### Content analysis

The interview transcripts were analysed using structuring content analysis [28, 29]. The objective of this method is to segregate transcripts into distinct manageable units (‘meaning units’). We used a deductive approach to coding. For this purpose, a coding tree with meta- and sub-codes was created before starting the analysis. If a meaning unit could not be successfully assigned to one of the pre-specified codes, the option of adding a new category in the coding tree was discussed. The respective decisions were made based on consensus between the two coders.

Meaning units referring to the meta-code ‘challenges in vertigo management in primary care’ were assigned to sub-codes according to the specific field of the challenge (‘diagnostics’, ‘therapy’, or ‘referral/health care system’). A fourth sub-code (‘patient-related challenges’) was added in the coding process.

Meaning units related to the meta-code ‘barriers and enablers of guideline-adherent care’ were further assigned to sub-codes structured according to the COM-B model (‘psychological capability’, physical capability’, ‘social opportunity’, ‘physical opportunity’, ‘automatic motivation’, ‘reflective motivation’). During the coding process, it turned out that the two types of motivation were frequently overlapping and thus hardly distinguishable. As a consequence, these two sub-codes were merged into one (‘motivation’).

Meaning units assigned to the meta-code ‘guideline expectations’ were sub-coded according to the domains of the AGREE II framework [30]: ‘Scope and Purpose’, ‘Stakeholder Involvement’, ‘Rigour of Development’, ‘Clarity of Presentation’, ‘Applicability’, and ‘Editorial Independence’.

For the meta-code ‘opinions and preferences regarding potential intervention methods’, sub-codes of the main EPOC intervention categories (*‘*professional’, ‘financial’, ‘organisational’, and ‘regulatory’ interventions) were assigned. The types of professional interventions were further differentiated with a second level of sub-codes. The meta-code ‘potential incentives’ was used without sub-codes. An additional sub-code (‘refusal of any type of intervention’) was added in the coding process.

A comprehensive description of the applied coding tree including meaning unit examples and definitions of all pre-defined meta- and sub-codes can be found in Additional file 2.

The directed content analysis was done independently by two researchers (AS and EK) with MAXQDA12 and subsequently synthesised. In case of divergent coding, coding was decided by consensus after discussion between the two researchers.

### Graphical representation of the results of the analysis

While it was originally planned to treat the various interview topics as separate analysis blocks, the coding of meaning units suggested that PCPs viewed these topics as considerably more interrelated. A post-hoc code co-occurrence model generated with the function MAXMAPS in MAXQDA12 mapped all codings which were simultaneously assigned to one meaning unit. In a subsequent step, each co-occurrence was checked for a logical relationship (i.e. one topic being perceived by the PCPs as a cause or consequence of the other), reducing the model to the meaningful connections. To summarise the perceived effects of guideline characteristics and intervention methods on vertigo and dizziness management in the primary care setting in a graphical representation, we re-structured this reduced code-co-occurrence model according to the COM-B model.

## Results

### Participant recruitment

Out of the 13 PCPs we contacted, 12 PCPs agreed to participate (response rate 92%). All 12 PCPs returned the QCPC questionnaire.

### Data collection

Seven telephone and five face-to-face interviews took place between December 2015 and February 2016 (nine by AS, two by EK, one by SU supervised by AP). The mean interview duration was 18 min (range 10–34 min). Five QCPC questionnaires were filled out by the PCP directly after the interview, one of those by telephone (as a standardised telephone interview). Eight questionnaires were sent back per mail.

### Pilot testing

Since the pilot testing supported the developed interview structure and did not result in any fundamental changes, the pilot interview was included into the main analysis.

### Characteristics of the PCPs and their practices

Of the 12 participating PCPs, eight were men and four were women. Mean age was 49 years (range 32–74) and mean time in practice was 14 years (range: 1–43). On average, participating PCPs consulted around 240 patients per week (range 75–500), and the number of patients with specific demands (multimorbidity, elderly, migration background, etc.) varied widely between practices (Table 1). All PCPs indicated that they used evidence-based guidelines for patient treatment at least sometimes.

**Table 1:** Practice and PCP characteristics based on the QCPC questionnaire

PCP characteristics	
*N*	12
Sex, *n* (%)	
Male	8 (67)
Age in years, mean (min–max)	49 (32–74)
Years in practice, mean (min–max)	14 (1–43)
Practice characteristics	
Community size	
Inhabitants, *n* (%)	
< 5000	6 (50)
5.000–20.000	1 (8)
> 20.000–100.000	1 (8)
> 100.000	4 (33)
Practice size	
Patients per week, mean (min–max)	240 (75–500)
Percentage of patient groups, mean (min–max)	
≥ 60 years	37 (20–70)
≥ 2 chronic diseases	48 (30–80)
Mental diseases	20 (5–50)
Migration background	12 (0–70)
Practice employees by profession, mean (min–max)	
PCPs	2.4 (1–6)
Assistants	3.6 (1–6)
Nurses	0.1 (0–1)
Apprentices	0.4 (0–2)
Other	0.4 (0–2)
Frequency of using evidence-based guidelines for patient treatment, *n* (%)	
Always	1 (8)
Most of times	4 (33)
Sometimes	7 (58)
Rarely	0
Never	0
Training	
Participation frequency in trainings in the last 12 months, mean (min–max)	
On medical topic (*N* = 12)	11 (3–40)
On practice organisation (*N* = 10)	0.7 (0–2)
Participation frequency in quality circles, mean (min–max)	3.3 (2–4)
Individual selection criteria for trainings	
Date	10 (83)
Content	11 (92)
Supporting program	1 (8)
Place	11 (92)
Speaker	6 (50)
CME points	2 (17)
Exchange with colleagues	4 (33)
Independence from the industry	6 (50)

### Analysis of the interviews

In total, 828 meaning units were coded. Of these, 66 meaning units were labelled with the meta-code ‘challenges in vertigo management in primary care’. The meta-code ‘barriers and enablers of guideline-adherent care’ was covered with 163 meaning units (meaning units per sub-code: ‘psychological capability’: *n* = 25, ‘physical capability’: *n* = 6, ‘social’ and ‘physical opportunity’: *n* = 23 and *n* = 46, respectively, ‘motivation’: *n* = 63), while further 20 meaning units were labelled ‘potential incentives’. The meta-code ‘guideline expectations’ was attributed to 100 meaning units, and ‘opinions and preferences regarding potential intervention methods’ to 194 meaning units.

#### Challenges in the management of vertigo and dizziness patients (COM-B aspect: behaviour)

PCPs reported four main challenging fields in their present vertigo management routine: (1) diagnostics, (2) therapy, (3) the health care system, and (4) patient-related challenges (for a comprehensive overview see Additional file 3).

The challenges related to diagnostics referred primarily to the unspecific vertigo and dizziness symptoms:

The main problem is that we often don’t know exactly what we are talking about, if a patient says he is dizzy. […] The patient describes a whole range of sensations with that […] (PCP4).

Also, PCPs stated that they missed standardised procedures, especially to identify red flags:

Having a little help […] where are the alarm signals, when do I have to react, what should I not miss, when can I rather wait and see. (PCP4).

While treatment options were perceived as straightforward in most situations, treatment of aged patients and patients with chronic symptoms posed problems:

You have to differentiate. For acute vertigo, it is not too difficult […]. For chronic vertigo it is difficult. And […] vertigo in older patients, that often is very resistant to treatment and that makes it difficult (PCP3).

Health care system-related challenges included the fragmented diagnostic process between primary care and different secondary care specialties. Also, PCPs noted a general lack of resources, including specialised services, and long delays in getting a specialist appointment:

The problem in vertigo management is, that with a referral, the vertigo is assigned to a certain specialty. And in case of doubt, the neurologist just notes that the vertigo is not related to his specialty. Period. The system leads to the fact that he does not think a step further: Where do we go from here? But instead he will just send the patient back. […] That means, that a crazy amount of time is lost through the recurrent returns of the patient to the PCP practice (PCP7).

But the problem in part also lies in the time and resources, i.e. you will automatically refer the patient, although you could also do everything by yourself, just for time and structural reasons (PCP7).

With regard to patient-related challenges, the PCPs reported patients’ limited readiness to participate in diagnostic processes, depending on symptom severity and comorbidities. Additionally, PCPs noted that patients were often keen on marketed (i.e. not evidence-based) medications. For some other patients, in contrast, over-the-counter payments for pharmacotherapy recommended by the PCP may result in adherence problems.

#### Barriers and facilitators of appropriate management of vertigo and dizziness patients and of successful guideline introduction: COM-B aspects: capability, opportunity, and motivation

PCPs reported difficulties in establishing the correct diagnosis due to lack of knowledge, skills, or experiences with vertigo-specific diagnostic tests:

Testing something which you haven’t learned […], I wouldn’t dare do that in the practice, and if I get into trouble that will cost me a lot of time (PCP1).

Additionally, the physical environment was considered a barrier for conducting the complete diagnostic process within the primary care setting because of time pressure, missing practice facilities, and perceived inadequate compensation for the required extra effort:

The most important barrier is time. Because, if a vertigo patient sits in front of you, usually 3,4,5,6,7,8,9,10 more patients with many other problems are waiting outside, such that you just don’t have the time and peace to dedicate yourself to the topic as much as the topic deserves it (PCP7).

[…] And the benefactors don’t cooperate in these cases anymore. […] The financial resources are also somewhat limited (PCP10).

Facilitators and barriers related to opportunities, such as the support of the practice team and exchange with colleagues were also mentioned:

of course it also depends on the support of the practice team (PCP3).

if you have a good quality circle, which works well on primary care topics, that is great, if you are able to involve them (PCP11).

Tertiary care centres dedicated to vertigo care, if available, were further seen as facilitating appropriate care.

Regarding barriers to implementing vertigo and dizziness guidelines, competing priorities were frequently mentioned, with other diseases being perceived as more relevant:

Once I picture the practice, I see 50 or 100 things in front of me, where we would need guidelines with algorithms which should be implemented. […] from a psychological perspective, you often tend to tackle the easy things. I mean, writing SOPs for bladder infections into your quality management system is far easier (PCP7).

[…] reasons for consultation […] are also perceived differently. […] Like palliative care, […] these topics have whole different emotional coverage, and you have to surpass these topics first (PCP7).

If the guideline was seen as criticism of the PCPs’ work, this was perceived as a motivational barrier to use it.

On the other hand, self-discipline and general organisational skills were rated as crucial for successful implementation. Social facilitators for guideline implementation such as the cooperation of practice team, patients, and the PCPs’ individual network of personally known specialists were also mentioned.

Factors increasing physical opportunity for guideline implementation were adequate staff numbers and electronic availability of knowledge resources.

The PCPs’ motivation to apply a certain guideline was rated higher if it increased self-confidence, knowledge, treatment success, simplified practice, related to a high-prevalence disease, offered protection in case of legal responsibility, enhanced the PCPs credibility towards patients, and was financially supported.

Further incentives were provision of instructions how to implement a guideline into practice and accompanying remote support in diagnosis for difficult cases.

#### Guideline expectations

Attitudes and expectations towards guidelines, illustrated by characteristic citations, are presented in Additional file 4.

PCPs perceived guidelines as poorly adapted to the everyday reality of the primary care setting:

Because one gets the impression that the colleagues who create the guidelines often don’t work in everyday practice anymore, under the pressures and impressions of doing the practical job (PCP2).

Guidelines were perceived as frequently unclear and too long. Diagnostic and therapeutic algorithms and one-page summaries would, according to the PCPs’ opinion, highly facilitate guideline use:

if you have to read through a 300-page guideline by yourself and extract something for yourself, that is always difficult and slowing you down. Thus, if you have short forms, if you have ready-made instruments to which you can revert, then obviously that is helpful (PCP4)

Patient information and self-help material and tools for involving the practice team in the case management process were seen as further support.

#### Potential target points for interventions and incentives resulting from the identified relevant COM-B aspects

The PCPs’ attitudes towards potential intervention approaches are displayed in detail in Additional file 5. Relatively few PCPs had already pre-established preferences. Among these, the most frequently preferred method was the educational meeting with interactive involvement.

After reading out potential intervention methods, the educational meeting and organisational interventions remained the preferred methods. Acceptance of patient-mediated interventions, reminders, marketing tools, and financial interventions was also high. Distribution of educational materials, local consensus process, and outreach visit were rated favourably, but to a slightly lesser extent. Acceptance of regulatory interventions and audit was balanced with equal pros and cons, while involvement of local opinion leaders and mass media were clearly refused.

**Fig. 1:**
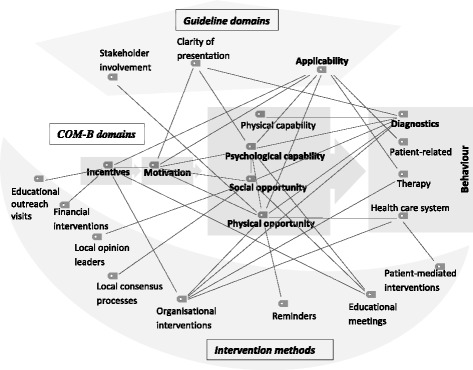
Associations of guideline aspects and intervention methods on COM-B model for vertigo management. Lines indicate aspects reported as interrelated by to the PCPs (code co-occurrences). Bold: the domains most stressed by the PCPs

Figure 1 shows which guideline characteristics and intervention methods were frequently mentioned in conjunction with statements belonging to the capability-opportunity-motivation triad of the COM-B model.

## Discussion

Our interviews with primary care physicians (PCPs) found that one main challenge in the management of vertigo and dizziness was to establish a definite diagnosis. Main reasons given were lack of opportunities for exchange and cooperation with colleagues, time and financial pressure, and lack of equipment. Improving diagnostic skills should thus be one primary objective. Guidelines were seen as both a potential additional burden and a potential facilitator, depending on their clarity, length, and applicability in everyday practice. PCPs seemed to be open for both educational meetings and organisational interventions. Based on these results, we will develop a tailored intervention to improve the quality of primary care for vertigo and dizziness patients.

The perceived challenges in establishing a diagnosis are well confirmed by other quantitative studies on vertigo: Comparing the referral diagnoses of PCPs with the established diagnoses of a tertiary level vertigo care centre [1], significant differences were detected, with PCPs frequently under-diagnosing certain types of vertigo, while the most frequent referral diagnosis was ‘unclear vertigo’. A similar study [2], analysing more than 2000 patients, found that most of them underwent multiple diagnostic procedures without receiving a definite diagnosis or appropriate treatment. This suggests current sub-optimal diagnosis of vertigo symptoms in primary care.

As in our study, limited resources in terms of time, finance, organisation and workforce [31], which frequently result in a perceived effort-reward imbalance [32], have been suggested as barriers. However, time barriers do not only derive from the work load of consultations but also from guidelines themselves, which have been found to demand unmanageable sets of tasks. For example, following the recommendations set for the ten most frequent chronic diseases would already exceed the time resources available in primary health care [33]. In our interviews, PCPs stressed the need for guidelines to be of help, to be applicable and clear, and not to be an additional burden. Likewise, several systematic reviews found that guidelines were too complex and complicated for use [34, 35].

To resolve this issue, educational meetings and interventions targeting management have been reported to achieve the highest effect on guideline adherence in primary care [36]. These two interventions were also mentioned as most effective in our study. This aligns well with the results of a recent survey carried out by the German College of General Practitioners and Family Physicians [37]. Integration of guideline principles into the practice management software was reported as a further facilitator both in our study and in the literature [37].

Lack of motivation for change has been repeatedly reported as a major barrier in the literature [31, 38]. Several authors hypothesise that lack of motivation is the main reason for low intervention effectiveness [13, 34, 39]. In our study, we were not able to detect major motivational issues; however, some of the reported barriers, e.g. time constraints, might be interpreted as a motivational barrier.

### Strength and limitations

This study offers first-hand in-depth insights into the perceived challenges of vertigo management and guideline use as well as intervention preferences from the perspective of the primary care providers, who are also the most promising targets for potential future interventions in the primary care setting to improve care for vertigo and dizziness patients. Another strength of this study lies in the broad theoretical basis which was applied to the development of the interview structure and guided the coding and analysis process. This explicit use of theoretical background, although it is a valuable quality feature of implementation research in primary care, is still far from being standard practice [18, 40]. There is no consensus in criteria selecting the most appropriate theory [41]. Merging relevant constructs from the determinant frameworks TDF and CFIR into one interview structure and organising these constructs according to the COM-B implementation framework of behaviour change gave us the confidence that we would not miss out on important aspects of vertigo and dizziness management in primary care. Also, theory represented a sound starting point for analysis (as the coding scheme could be based on it and definitions of certain constructs such as motivation were available from the literature). Furthermore, having explicitly based our study on theory will provide the possibility to compare our results with those of other theory-based studies. Our results indicate that, by changing capability, opportunity, and motivation, well-designed guidelines and supporting interventions may improve PCPs’ management of vertigo and dizziness patients. The specific pathways can be graphically depicted and any subsequent implementation trial can be evaluated using the same theoretical model.

Still, the following limitations should be kept in mind: First, we recruited PCPs from a list of PCPs who had already shown interest in participating in another vertigo-related study of our working group. Thus, as every research project which requires participant engagement, this study bears some risk of selection bias because the sample is likely to consist of highly engaged PCPs who were already sensitised to the topic. Thus, we might even have underestimated the problems encountered in the management of vertigo and dizziness in primary care. In addition, with 12 interviews in total, the sample size is rather small. However, the physicians included were diverse in their characteristics, so, with information saturation achieved, we are confident that a broad range of relevant aspects was covered. This is in line with literature suggesting that, for theory-based interview studies, around 13 interviews are usually sufficient to achieve saturation [42]. This criterion, which, though originally developed for interview studies based on the Theory of Planned Behaviour, has also been successfully used in qualitative studies based on the TDF [43, 44].

Due to pragmatic reasons, e.g. practices situated in rural areas, and PCPs’ time preferences, we decided to conduct some of the interviews by telephone. It is sometimes argued that the quality levels between face-to face and telephone interviews may vary [45]. Indeed, our face-to-face interviews took on average 6 min longer than those conducted via telephone, suggesting a greater readiness of the PCPs to share detailed experiences in a face-to-face encounter. At the same time, our impression as interviewers was that both telephone and face-to-face interview participants did not hesitate to share criticism regarding guideline usability and generally provided the same richness of information.

We cannot completely rule out response bias by social desirability. However, PCPs were generally frank in their criticisms and very open about their problems; therefore, we consider the risk for bias rather low. Still, lack of skills as well as negative opinions towards guideline implementation and intervention methods may be even more widespread than can be inferred from our results.

Also, as our objective was to gain comprehensive information rather than agreement, and no difference was made with regard to minor or major themes in the analysis, one has to keep in mind that the presented PCPs’ opinions and experiences may not have been shared by all of them, but rather cover a broad range of existing experiences.

## Conclusions

Vertigo management in the primary care setting poses several challenges to PCPs, especially in the field of diagnostics. Perception of both their own capabilities and opportunities highly influence the PCPs’ subsequent engagement in vertigo and dizziness care. Our results indicate that guideline implementation should be supported through educational meetings and organisational change. Also, authors of guidelines should verify that the proposed actions fit into PCPs’ daily routine or, better still, improve daily routine. This is of particular relevance for the management of vertigo and dizziness where PCPs are dissatisfied with the current situation. Guideline implementation may then contribute to more effective diagnosis and treatment in the primary care setting and ultimately increase patient well-being.

## Additional files

Additional file 1: Interview structure, including the original German set of questions and the English translation. This file includes the original German interview structure as well as an English translation. This interview structure was developed for qualitative interviews with PCPs based on TDF, CFIR, and COM-B as theoretical foundations. The interview structure additionally includes prompts based on the EPOC list of interventions. (DOCX 61 kb)

Additional file 2: Codebook of content analysis. This codebook contains definitions of meta-codes and sub-codes according to which the interview meaning units were to be clustered. In addition, examples for meaning units are given for each meta-code and the questions which were expected to trigger responses belonging to each meta-code are listed. (DOCX 80 kb)

Additional file 3: COM-B aspects of vertigo management in the primary care setting. This table lists content analysis results including English translations of exemplary citations for the COM-B aspects of vertigo management in the primary care setting. (DOCX 56 kb)

Additional file 4: PCPs’ guideline expectations in vertigo management. This table lists English translations of characteristic citations for the PCPs’ view about guideline-related facilitators and barriers grouped according to the AGREE II. Framework. (DOCX 47 kb)

Additional file 5: PCPs’ opinion about intervention methods. This table lists English translations of characteristic citations for the PCPs’ view on various intervention methods grouped according to the EPOC list of interventions. (DOCX 49 kb)

## Abbreviations

AGREE II: Appraisal of Guidelines for Research & Evaluation
CFIR: Consolidated Framework for Implementation Research
COM-B: The triad of capability, opportunity, and motivation for behaviour change
EPOC: Cochrane Effective Practice and Organisation of Care Group
MD: Medical doctor
MPH: Master of Public Health
PCP: Primary care provider
PhD: Doctor of Philosophy
QCPC: Questionnaire of Chronic Illness Care in Primary Care
TDF: Theoretical Domains Framework
