# Correction to: barriers and facilitators for the management of vertigo: a qualitative study with primary care providers

**DOI:** 10.1186/s13012-018-0776-z

**Published:** 2018-06-15

**Authors:** Anna-Janina Stephan, Eva Kovacs, Amanda Phillips, Jörg Schelling, Susanne Marlene Ulrich, Eva Grill

**Affiliations:** 1 Institute for Medical Information Processing, Biometry and Epidemiology, Ludwig-Maximilians-Universität München, Marchioninistraße 17, 81377 Munich, Germany; 2 German Centre for Vertigo and Balance Disorders, University Hospital, Ludwig-Maximilians-Universität München, Munich, Germany; 3 Institute for General Practice and Family Medicine, University Hospital, Ludwig-Maximilians-Universität München, Munich, Germany; 4 Munich Centre of Health Sciences, Ludwig-Maximilians-Universität München, Munich, Germany

## Correction

After publication of the original article [1] it was brought to the authors’ attention that a sentence was missing in the acknowledgement section. The full acknowledgement is included in this Correction article:
