# Supplementary material for: Barriers and facilitators for the management of vertigo: a qualitative study with primary care providers
Source: Implement Sci. 2018 Feb 8;13:25. doi: 10.1186/s13012-018-0716-y (PMC5806383; doi:10.1186/s13012-018-0716-y)
Supplement: Supplementary file 1 — Interview structure, including the original German set of questions and the English translation. This file includes the original German interview structure as well as an English translation. This interview structure was developed for qualitative interviews with PCPs based on TDF, CFIR, and COM-B as theoretical foundations. The interview structure additionally includes prompts based on the EPOC list of interventions. (DOCX 61 kb) [file 13012_2018_716_MOESM1_ESM.docx]

Additional files

Additional file 1. Interview structure, including the original German set of questions and the English translation.

| **English translation** | **Original German set of questions** |
| --- | --- |
| Explanation:   - grey fields are instructions for the interviewer - *Italics: connection or explanatory text* - Underlined text: Main question - Listing: supplementary questions (to be used only if the spontaneous response has not contained any concrete information) | Erklärung:   - graue Felder gelten als Anweisung für den Interviewer - *Kursivschrift: Verbindungs- oder Erklärungs-Text* - unterstrichener Text: Hauptfrage - Auflistung: Ergänzungsfragen (sie sind nur dann anzuwenden, wenn die spontane Antwort keine konkrete Information enthalten hat) |
| Introduction: thanking, warm-up  *I would like to express the acknowledgement on behalf of our team and stress how grateful we are for your participation in this interview and in the cohort study in general. The aim of the study is to improve the care of vertigo.*  *The fact that you share your practical knowledge with us highly contributes to this. If you wish, we will gladly inform you about the final results as soon as the follow-up has been completed.*  I ask for your permission to make a voice recording, not to miss any of your comments. The transcription of the recording and the evaluation will be carried out in an anonymous form, and subsequently cannot be traced back to you. | Einleitung: Bedanken, Aufwärmen  *Ich möchte mich auch im Namen unseres Teams ganz herzlich für Ihre Teilnahme an diesem Interview und der Kohortenstudie im Allgemeinen bedanken. Ziel der Studie ist die Verbesserung der Versorgung von Schwindelerkrankungen. Dass Sie Ihr Praxiswissen mit uns teilen, leistet dazu einen wichtigen Beitrag. Wenn Sie möchten, werden wir Sie gern über die endgültigen Resultate informieren, sobald das Follow-up abgeschlossen ist.*  Ich bitte um Ihre Zustimmung, eine Sprachaufnahme zu machen, damit wir keine Ihrer Anmerkungen vergessen. Die Abschrift der Aufnahme und die Auswertung werden in anonymisierte Form durchgeführt, und können danach nicht mehr mit Ihrer Person in Verbindung gebracht werden. |
| *The aim of this interview is to get a more sophisticated understanding about the aspects of vertigo management with respect to the primary care setting, and to provide you the possibility to freely express your view which wasn`t covered by the questionnaire.*  1. Question - Exploring the experiences concerning the study  Would you first tell me about your experience in the context of the cohort study?  Supplementary questions if needed for warming up:   - Was the test battery acceptable for you, or do you have any recommendations for improving the questionnaires? If so, what exactly? - How much were you and your team burdened? - How easy or difficult have you found the recruitment of the patients? Why? | *Das Ziel dieses Interviews ist es, ein detaillierteres Verständnis der Einflussfaktoren für Schwindelversorgung in der Hausarztpraxis zu entwickeln. Dabei ist es uns wichtig, Ihnen die Möglichkeit zu bieten Ihre Sichtweise, die noch nicht durch den Fragebogen abgedeckt wurde, frei zu äußern.*  1. Frage - Erfahrungen hinsichtlich der Studie erkunden  Würden Sie mir zunächst über Ihre Erfahrungen im Rahmen der Kohortenstudie berichten?  Ergänzungsfragen, falls zur Aufwärmung benötigt:   - Waren die Fragenbögen für Sie akzeptabel, oder haben Sie irgendwelche Empfehlungen zur Verbesserung der Umfrage? Falls ja, was genau? - Wie stark waren Sie und Ihr Team dadurch belastet? - Fanden Sie die Rekrutierung der Patienten leicht oder schwer? Warum? |
| 2. question - Identification of gaps between evidence and practice and the necessary changes in behaviour, its barriers as well as facilitators to reduce these gaps  How easy or difficult do you find it to manage vertigo patients? Why?  Supplementary questions if few concrete information was communicated:   - What important aspects of dizziness management have you detected, either from your perspective or from the patient`s? - Do you think you should change something in your methodology? - What supportive factors and barriers exist here? - What would you describe as the main motivator or main obstacle, either from your side or from an organizational standpoint? - What could make this task easier for you? | 2. Frage – Identifizierung der Lücken zwischen Evidenz und Praxis und der notwendigen Verhaltensänderungen sowie diesbezüglicher Barrieren und unterstützender Faktoren, um diese Lücken zu verringern  Wie leicht oder schwer finden Sie es, Schwindelpatienten zu behandeln? Warum?  Ergänzungsfragen, falls wenig konkrete Informationen mitgeteilt wurden:   - Welche wichtigen Aspekte des Schwindelmanagements sind Ihnen aufgefallen, entweder aus Ihrer Perspektive oder aus der des Patienten? - Glauben Sie, dass Sie etwas in Ihrer Methodik verändern sollten? - Welche unterstützenden Faktoren und Barrieren existieren dabei? - Was würden Sie als wichtigsten Motivator oder wichtigstes Hindernis bezeichnen, entweder von Ihrer Seite aus oder aus organisatorischer Sicht? - Was könnte diese Aufgabe für Sie erleichtern? |
| Transition to the Guideline  *Perhaps you know that a S1 guideline of vertigo diagnosis and therapy is already available. This was created by the German Society of Neurology. An S3 guideline, possibly better adapted to the needs of family practice, is drafted currently by the German Society of General Medicine and Family Medicine. S3 guidelines are also based on a higher level of evidence than S1 guidelines. The publication of this guideline is planned for 2016.* | Überleitung zu der Leitlinie  *Vielleicht wissen Sie, dass bereits eine S1-Leitlinie über Schwindel-Diagnose und –Therapie verfügbar ist. Diese wurde von der Deutschen Gesellschaft für Neurologie erstellt. Eine möglicherweise besser an die Bedürfnisse der Hausarztpraxis angepasste S3-Leitlinie wird aktuell von der* ***Deutschen Gesellschaft für Allgemeinmedizin und Familienmedizin ausgearbeitet. S3-Leitlinien beruhen zudem auf einem höheren Evidenzgrad als S1-Leitlinien. Die Veröffentlichung dieser Leitlinie ist*** *für 2016 geplant.* |
| 3. Question - knowledge and attitudes regarding the vertigo-guideline  What is your opinion with regard to these vertigo guidelines?   - What do you think about the content, the quality and reliability of the currently available S1 dizziness guideline? - How do you rate their relevance in your practice? - What do you expect from the DEGAM guideline? | 3. Frage – Kenntnisse und Einstellungen hinsichtlich der Schwindel-Leitlinie  Welche Meinung haben Sie im Hinblick auf diese Schwindel-Leitlinie?   - Was denken Sie über den Inhalt, die Qualität und Zuverlässigkeit der derzeit verfügbaren S1-Schwindel-Leitlinie? - Wie bewerten Sie deren Relevanz in Ihrer Praxis? - Was erwarten Sie von der DEGAM-Leitlinie? |
| Transition to the intervention-supported implementation  *Ideally, a new guideline is not just published but their implementation is supported by intervention measures. Next we would like to learn your opinion on the new vertigo-guideline and its intervention-based implementation.* | Überleitung zu der Intervention-unterstützte Umsetzung  *Idealerweise wird eine neue Leitlinie nicht einfach nur veröffentlicht, sondern deren Umsetzung mit Interventionsmaßnahmen unterstützt. Im Folgenden möchten wir Ihre Meinung über eine neue Schwindel-Leitlinie und über deren Interventions-gestützte Umsetzung erfahren.* |
| 4. Question - Exploration of the factors influencing the implementation (1): Capability  (the domains of these field: knowledge, scientific rationale, beliefs, memory, attention, decision process, procedural knowledge, behaviour regulation, skills, self-efficacy)  Do you think you would be able to introduce and maintain following vertigo guidelines in your practice? | 4. Frage - Erforschung der Einflussfaktoren der Umsetzung (1): Fähigkeit  (die Domänen dieser Bereiche: Wissen, wissenschaftliche Begründung, Vorstellungen, Gedächtnis, Aufmerksamkeit, Entscheidungsprozess, prozedurales/verfahrenstechnisches Wissen, Verhaltensregelung, Fähigkeiten, Selbstwirksamkeit)  Glauben Sie, dass Sie in der Lage wären, eine Schwindel-Leitlinie einzuführen und nachhaltig in Ihrer Praxis zu verfolgen? |
| Additional questions if the answer was not exhaustive:   - What would support or prevent you from doing so? - How do you estimate the difficulties of following the guideline? Why? How does it fit into your present practice? - What circumstances could support the implementation of the guideline in your practice? - Are you satisfied with the process of introducing the guideline? Why? - Do you find any of the above aspects relevant for you and your setting? Why? | Weitere Fragen, wenn die Antwort nicht vollständig war:   - Was würde Sie darin unterstützen oder behindern? - Wie schätzen Sie die Schwierigkeiten ein, einer Schwindel-Leitlinie zu folgen? Warum? Wie passt es zu Ihrer momentanen Praxis? - Welche Umstände könnten die Einführung der Leitlinie in Ihrer Praxis unterstützen? - Sind Sie mit dem Prozess der Einführung von Leitlinien zufrieden? Warum? - Finden Sie eines oder mehrere der obengenannten Probleme relevant für Sie und Ihre Praxis? Warum? |
| 5. Question - Exploration of the factors influencing the implementation (2): Opportunity  (domains of this field: social influences and norms, peers and opinion leaders, patient needs, resources)  How do you rate the chance of implementing vertigo guidelines in your setting? Why? What are the main influencing factors and required resources? | 5. Frage - Erforschung der Einflussfaktoren der Umsetzung (2): Gelegenheit  (die Domänen dieser Bereiche: soziale Einflüsse und Normen, Kollegen und Meinungsführer, die Bedürfnisse der Patienten, Ressourcen)  Wie bewerten Sie die Möglichkeiten, die Schwindel-Leitlinie in Ihrer Praxis zu implementieren? Warum? Was sind die wichtigsten Einflussfaktoren und die benötigten Ressourcen? |
| Additional question if the answer was not exhaustive:   - What about the contribution of your peers and organisational background? - How do you assess the impact of your corporate culture (general beliefs, values, assumptions) regarding the implementation of the intervention? - To what extent do these social/professional influences facilitate or hinder introducing vertigo guidelines? - Who are these stakeholders, whose opinion do you find the most relevant regarding this issue? - What do these influential people think about the guideline and the intervention? - How is your peer network positioned in this question? - Do you find any of the above aspects relevant for you and your practice? Why? | Weitere Fragen, wenn die Antwort nicht vollständig war:   - Wie ist der Beitrag Ihrer Kollegen und der organisatorische Hintergrund? - Wie beurteilen Sie den Einfluss Ihrer Unternehmenskultur (allgemeine Vorstellungen, Werte, Annahmen) in Bezug auf die Umsetzung einer Intervention? - Inwieweit erleichtern oder behindern diese sozialen / beruflichen Einflüsse die Einführung der Schwindel Leitlinie? - Wer sind die Stakeholder, deren Meinung Sie zu diesem Thema am relevantesten finden? - Was halten diese einflussreichen Personen von der Leitlinie und der Intervention? - Wie ist Ihr Peer-Netzwerk in dieser Frage aufgestellt? - Finden Sie irgendwelche der obengenannten Probleme relevant für Sie und Ihre Praxis? Warum? |
| Question 6 - Exploring the factors influencing the implementation (3): Motivation  (domains of these field: emotions, professional role and identity, intentions, beliefs)  Is adhering to the vertigo guideline compatible or in conflict with your professional standards/identity? | 6. Frage - Erforschung der Einflussfaktoren der Umsetzung (3): Motivation  (die Domänen dieser Bereiche: Emotionen, professionelle Rolle und Identität, Absichten, Annahmen)  Ist die Anpassung an die Schwindel Leitlinie vereinbar oder steht sie in Konflikt mit Ihren professionellen Standards / Ihrer Identität? |
| Additional question if the answer was not exhaustive:   - What feelings do you have in this regard? - Do you anticipate any problems with following vertigo guidelines? - What are the benefits and disadvantages of introducing the guideline for you? | Weitere Fragen, wenn die Antwort nicht vollständig war:   - Welche Gefühle haben Sie diesbezüglich? - Sehen Sie irgendwelche Probleme voraus, der Schwindel Leitlinie zu folgen? - Was sind die Vorteile und Nachteile der Einführung der Leitlinie für Sie? |
| 7. Question - Strategy and incentives  What incentives would influence your decision to implement and follow the guideline?  Would you prefer financial or other incentives? | 7. Frage - Strategie und Anreize  Welche Anreize würden Ihre Entscheidung, die Leitlinie umzusetzen und zu befolgen, beeinflussen?  Würden Sie finanzielle oder andere Anreize bevorzugen? |
| 8. Question - Preferences regarding the intervention  What form of guideline implementation would you prefer? | 8. Frage – Präferenzen hinsichtlich der Intervention  Welche Interventionen zur Unterstützung der Leitlinienimplementierung würden Sie bevorzugen? |
| If a decisive answer is provided, then ask:   - How would you characterise this method (i.e. how long, how many times, by whom and by what means, depending on what was mentioned)? - Is there any other additional method which you would find supportive and that fits into your practice? - Do you think that the most acceptable form of implementation is also the most effective? | Wenn eine bestimmte Antwort gegeben wurde:   - Wie würden Sie diese Methode präzisieren (z.B. wie lange, wie oft, von wem und wodurch, je nachdem, was genannt wurde)? - Gibt es eine andere zusätzliche Methode, die Sie hilfreich fänden, und die in Ihre Praktik/Methodik und Ihren Zeitrahmen passt? - Glauben Sie, dass die am häufigsten verwendete Form der Umsetzung gleichzeitig auch die effektivste ist? Was sind die grundlegenden Aspekte? |
| If no clear answer/preference is provided, then list the main methods of intervention:  I would now like to mention a few methods, please let me know which you prefer. Please interrupt me when I say a method that you find helpful.   - Distribution of educational materials - Educational meetings (which form do you prefer, e.g. personal, e-learning...) - Local consensus processes in terms of content and implementation of the guideline - Educational outreach visits/ counselling by experts in your practice - Involvement of Local opinion leaders - Patient mediated information - Audit and feedback - Reminders(electronically (e.g. Apps), by phone, in writing) - Measures to change existing attitudes and perspectives - Use of mass media to increase the awareness of the problem | Wenn keine klare Antwort / Präferenz mitgeteilt wurde, dann listen die wichtigsten Methoden der Intervention:  Ich möchte Ihnen nun einige Methoden nennen, lassen Sie mich bitte wissen, welche Sie bevorzugen. Bitte unterbrechen Sie mich, sobald ich eine Methode nenne, die Sie als hilfreich empfinden.   - Verteilung von Informationsmaterialien - Fortbildungen (Welche Form bevorzugen Sie, z.B. persönlich, e-Learnings…) - Lokale Konsensprozesse hinsichtlich des Inhalts und der Einführung der Leitlinie - Besuche / Beratungen durch Experten vor Ort in Ihrer Praxis - Einbindung lokaler Meinungsführer - Patienten vermittelte Informationen - Audit und Feedback - Erinnerungen (elektronisch (z.B. Apps), telefonisch, schriftlich) - Maßnahmen zur Veränderung bestehender Einstellungen und Sichtweisen - Nutzung von Massenmedien zu Steigerung das Problembewusstseins |
| In addition to these professional interventions, there are also other approaches to support the implementation of guidelines. What methods would you prefer here? Please interrupt me again as soon as you find a method as effective. | Neben diesen Professionellen Interventionen gibt es natürlich auch weitere Ansatzpunkte zur Unterstützung der Implementierung von Leitlinien. Welche Methoden würden Sie hier bevorzugen? Bitte unterbrechen Sie mich wieder, sobald Sie eine Methode als zielführend empfinden. |
| - Financial intervention - Organisational interventions in the primary care setting - Structural measures in the health care system - Regulatory interventions | - Finanzinterventionen - Organisatorische Maßnahmen in der Hausarztpraxis - Strukturmaßnahmen im Gesundheitssystem - Regulatorische Interventionen |
| So I am finished with my questions from my side.  Is there anything else that is relevant to your view of which we have not spoken and which you would like to comment on?  I would like to take this opportunity to thank you once again for your time and for the valuable information you have provided to us. | Damit bin ich mit meinen Fragen am Ende angelangt.  Gibt es noch etwas auch Ihrer Sicht relevantes, über das wir nicht gesprochen haben und das Sie ergänzen möchten?  Dann möchte ich mich an dieser Stelle noch einmal ganz herzlich für Ihre Zeit und die wertvollen Informationen bedanken, die Sie uns zur Verfügung gestellt haben. |
| General supportive questions:   - Why do you think so? - I thank you for mentioning this aspect. May I ask you to consider other aspects as well? - What other aspects do you find important? | Allgemeine Hilfsfragen:   - Warum denken Sie so? - Ich danke Ihnen, dass Sie diesen Aspekt genannt haben. Darf ich Sie bitten, noch weitere Aspekte zu bedenken? - Welche anderen Aspekte halten Sie für wichtig? |
