# Supplementary material for: Barriers and facilitators for the management of vertigo: a qualitative study with primary care providers
Source: Implement Sci. 2018 Feb 8;13:25. doi: 10.1186/s13012-018-0716-y (PMC5806383; doi:10.1186/s13012-018-0716-y)
Supplement: Supplementary file 2 — Codebook of content analysis. This codebook contains definitions of meta-codes and sub-codes according to which the interview meaning units were to be clustered. In addition, examples for meaning units are given for each meta-code and the questions which were expected to trigger responses belonging to each meta-code are listed. (DOCX 80 kb) [file 13012_2018_716_MOESM2_ESM.docx]

Additional files

Additional file 2. Codebook of content analysis.

# General remarks

The codebook follows closely and more in-depth the theoretical background applied in the construction of the interview structure. The definitions of the aspects are cited from the referred literature either word-by-word or in a shortened form.

Meaning units were systematically extracted and assigned to the appropriate codes. Though the codebook was prepared prior to the start of the coding, in the course of the coding process, the coding guideline may have been refined by adding sub-codes, further differentiating them in a more meaningful way, or removing them. The final consensus of the double coding of the meaning units was based on a finalised version of the codebook below.

# Warm-up: mapping experiences regarding the community cohort study

- definition / general description of the cluster: this question served as introduction and warming up, bridging the PCPs previous experiences with the planning of intervention. The information gained here, characterizing the PCPs attitudes regarding the cohort study and revealing difficulties will be helpful in fine-tuning the planned intervention study from technical aspects; however, doesn`t fit directly in the purpose of the present analysis and will not be published here.

### Referring Interview part

| The aim of this interview is to get a more sophisticated understanding about the aspects of vertigo management with respect to the primary care, and to provide you a possibility freely expressing your view which wasn`t covered by the questionnaire.  *1. Question - Exploring the experiences concerning the study*  Main question: Would you **at** first tell me about your experience in the context of the cohort study**?**  Supplementary questions if needed:   - Was the test battery acceptable for you, or do you have any recommendations for improving the questionnaires? If so, what exactly? - How much were you and your team burdened? - How easy or difficult have you found the recruitment of the patients? Why? |
| --- |

### Factors belonging to this cluster:

##### GPs` aspects

- - definition / description: all those factors which refer to the PCP in person, their role, their own activity concerning the recruitment, opinion, feeling, attitude
  - example:

"One forgets to include patients (...) one could have included many more patients. This is really burdenful and annoying "

"That's up to me, it's because I have to think about it and the daily routine of the practice prevents me to make the effort"

"The main problem is actually, that when there is a rush and operation is very high, then I don`t think about the study"

##### Patients` commitment

- - definition / description: experiences regarding the patients` attitudes, concerns, compliance
  - example:

"They were afraid of the study. And they have found everything too complicated, the questions."
"One shouldn`t make so much bureaucracy, shouldn´t provide too much information, that is, all the compulsory parts an ethical committee establishes, these are frightening for the patients and then it is more discouraging than encouraging to participate in the study."

##### Fitting to the praxis

- - definition / description: further aspects of feasibility among everyday-circumstances, time requirement, involvement/opinion/role of other primary care team members
  - example:

"It was not a burden"
"My team is not burdened by this, because I personally have done it"
"An assistant of mine has done it, she has filled out the questionnaires (...) (burdened) actually less. So they did this during the non-consulting time, which was fine "

##### Test battery

- - definition / description: opinions about the questionnaire (characteristics, content, feasibility, length)
  - example:

"They were good"
" In my view, the questionnaires are adapted well to the daily routine of the practice, they are fitting "
"They are acceptable, I find them OK"

##### Else

# Identifying challenges, barriers and facilitators of guideline-adherent vertigo care

- definition / general description: this section follows the structure of the Behaviour Change Wheel[[1](#_ENREF_1)] (Capability, Opportunity, Motivation – Behaviour; COM-B:). The COM structure is filled with the domains of the Theoretical Domains Framework [[2](#_ENREF_2)] (TDF) and, as completion, with the domains of the Consolidated Framework For Implementation Research[[3](#_ENREF_3)] (CFIR). The TDF covers/integrates mainly psychological theories; the CFIR collects the input from implementation research theories.

*(Remark: the first version of the interview structure listed all the above domains and their definition; however, time constrain led to a practical simplification, preserving the level of COM-subcategories.)*

## Behaviour: challenges in vertigo management

- definition / general description of the cluster: the current practice of the vertigo management has to be characterized, with the aim of identifying the weaknesses that should be improved.

### Referring Interview part

| *2. question - Identification of gaps between evidence and practice and the necessary changes in behaviour, its barriers as well as facilitators to reduce these gaps*  Main question: **How easy or difficult do you find to manage vertigo patients? Why?**  Supplementary questions if few concrete information was communicated:   - What important aspects of vertigo management have you detected, either from your perspective or from the patient`s? - Do you think you should change something in your methodology? - What supportive factors and barriers do exist here? - What would you describe as the main motivator or main obstacle, either from your side or from an organizational standpoint? - What could make this task easier for you? |
| --- |

### Factors belonging to the cluster:

### Main label: professional field of the problem

##### diagnostics

- - definition / description: the PCP reported problems regarding the diagnostics of vertigo
  - example:

„I see potential for improvement here, because I don’t have a standardised program. It is all still very intuitive when it comes to diagnostics.“

##### therapy

- - definition / description: the PCP reported problems regarding the therapy of vertigo
  - example:

„For acute vertigo, it is not too difficult because the treatment regimen is relatively clear. For chronic vertigo it is difficult. And especially, vertigo in older patients, this is often very, very resistant to treatment, and that is difficult.“

##### referral/health care system

- - definition / description: the PCP reported problems caused by the health care system
  - example:

„The problem in vertigo management is, that with a referral, the vertigo is assigned to a certain specialty. And in case of doubt, the neurologist just notes that the vertigo is not related to his specialty. Period. The system leads to the fact that he does not think a step further: Where do we go from here? But instead he will just send the patient back. And the same goes for the ENT specialist, cardiologist, angiologist and so on. That means, that a crazy amount of time is lost through the recurrent returns of the patient to the PCP practice, just because we have a system which demands and promotes the narrowmindedness of each and every specialty. Say, I do my part, I check on my part, and if everything is fine there, I am done. And that, of course, is problematic for the patient.“

##### patient-related

*Remark: this category was added during the coding process.*

- - definition / description: all aspects that refer to the patient`s characteristics, behaviour or attitudes, influencing his compliance towards the best case management
  - example:

“If the patient is not very mobile“

### Double label: structure of the above aspects

##### evidence-practice gap

- - definition / description: identifying the cause of the problem (i.e. of the evidence-practice gap)

##### what/who has to change

- - definition / description: to identify the possible target points for intervention, i.e. the change what is required in favour of achieving (or at least approximating) the ideal solution and/or the stakeholders who have the competence to influence above change (including the PCP himself, if he is the competent stakeholder in deciding to perform the change; i.e. he could make it independently if he wanted)

##### facilitators of this change

- - definition / description: all those actors/circumstances which have a positive influence on the aforementioned stakeholders/circumstances in favour of achieving the targeted change

##### barriers of this change

- - definition / description: all those actors/circumstances which have a negative influence on the aforementioned stakeholders/circumstances, thus prevent achieving the targeted change

## Capability

- definition / general description of the cluster: This aspect describes the psychological or physical ability to enact the required favourable behaviour.

### Referring Interview part

| *4. Question - Exploration of the factors influencing the implementation (1): Capability*  (the domains of these field: knowledge, scientific rationale, beliefs, memory, attention, decision process, procedural knowledge, behaviour regulation, skills, self-efficacy)  Main question: **Do you think you would be able to introduce and maintain following vertigo guidelines in your praxis?**  Additional questions if the answer was not exhaustive:   - What would you support or prevent you doing so? - How do you estimate the difficulties of following the guideline? Why? How does it fit into your present practice? - What circumstances could support the implementation of the guideline in your praxis? - Are you satisfied with the process of introducing the guideline? Why? - Do you find any of the above aspects relevant for you and your setting? Why? |
| --- |

#### psychological capability

- - definition / description: this factor covers the intellectual ability to engage in the necessary thought processes as comprehension or reasoning; includes knowledge management, i.e. understanding, learning and memorizing facts (e.g. scientific rationale and procedural knowledge), focusing attention, making good decisions, controlling own actions, regulating and modifying own behaviour.
  - example:

“ I don’t think that guidelines should substitute thinking“

„You have to continue your education. You have to be up to date.“

#### physical capability

- - definition / description: this factor refers to physical/practical skills (“an ability or proficiency acquired through practice”), strength and stamina.
  - example:

„Sonography of the carotid artery, for example, that we cannot do, we are not trained for that.“

## Opportunity

- definition / general description of the cluster: physical and social environment that influences (enables, facilitates or hinders) the behaviour

### Referring Interview part

| *5. Question - Exploration of the factors influencing the implementation (2): Opportunity*  [domains of this field: social influences and norms, peers and opinion leaders, patient needs, resources]  Main question: **How do you rate the chance of implementing vertigo guidelines in your setting? Why? What are the main influencing factors and required resources?**  Additional question if the answer was not exhaustive:   - What about the contribution of your peers and organisational background? - How do you assess the impact of your corporate culture (general beliefs, values, assumptions) regarding the implementation of the intervention? - To what extent do these social/professional influences facilitate or hinder introducing vertigo guidelines? - Who are these stakeholders, whose opinion do you find the most relevant regarding this issue? - What do these influential people think about the guideline and the intervention? - How is your peer network positioned in this question? - Do you find any of the above aspects relevant for you and your praxis? Why? |
| --- |

#### social opportunity

- - definition / general description: interpersonal influences, social cues and cultural norms that influence the way of thinking or behaving, e.g. social support, social/group norms and values, organisational development/climate , management, hierarchical or informal opinion leaders, team working, group conformity, peer pressure, management commitment, supervision, champions of change, networking and communication.
  - example:

„if you have a good quality circle, which works well on primary care topics, that is great, if you are able to involve them.“

#### physical opportunity

- - definition / general description: circumstances of a person’s situation or environment that influences the person`s behaviour or expected behaviour change or the development of skills and abilities. It includes availability of resources or channels of work- and information flow, locations, time, environmental stressors, or other characteristics of task environment.
  - example:

“The most important barrier is time.”

„The financial resources are also somewhat limited.“

## Motivation

- definition / general description of the cluster: reflective and automatic mechanisms that activate or inhibit behaviour

*Remark: during the analysis, the subcategories of automatic and reflective motivation were merged, as a result of partial overlapping (i.e. the currently automatic motivation may be rooted in an earlier reinforcement).*

### Referring Interview part

| *Question 6 - Exploring the factors influencing the implementation (3): Motivation*  (domains of these field: emotions, professional role and identity, intentions, beliefs)  Main question: **Is adhering to the vertigo guideline compatible or in conflict with your professional standards/identity?**  Additional question if the answer was not exhaustive:   - What feelings you have in this regard? - Do you anticipate any problem in following vertigo guidelines? - What are the benefits and disadvantages of introducing the guideline for you? |
| --- |

#### automatic motivation

- - definition / general description: emotions and impulses that arise from intrinsic drive states, desires, internal or innate dispositions; and habits resulting from associative learning and physiological states (e.g. positive/negative affect, stress, fear, burn-out, anxiety/depression, enjoyment). These processes are not connected to the present circumstances, don´t have any object (reward or punishment) in the current environment (however, they may be rooted in environmental influences of the past, e.g. primary or professional socialization, but being out of the scope of conscious present decisions).
  - example:

„And for the rest, it is just endogenous motivation, just to say, we want that, we want that.“

„Of course, if I look back after the implementation, and say: „look at this, we have made it,“ that is great, then you are happy, that motivates you, but the inner temptation beforehand is huge.“

#### reflective motivation

- - definition / general description: reflective processes which answer on environmental facts/changes, i.e. have in the environment a cause and/or an object to be achieved (receiving a reward or avoiding a punishment”). They are conscious intentions (“a conscious decision to perform a behaviour or a resolve to act in a certain way”) directed towards a goals ( “mental representations of outcomes or end states than an individual wants to achieve”) e.g. evaluations, decisions and plans, outcome expectancies, beliefs about own competence, self-efficacy, perceived behavioural control; individual stage of change.
  - example:

„ In the long run, we want to gain something from it […]. In the long run I save time, in the long run I have more satisfied patients, more satisfied medical assistants, more satisfied doctors, great structure, great quality management”

## **Incentives**

### Referring Interview part

| *Question8. - Strategy and incentives*  Main question: What incentives would influence your decision to implement the guideline and follow**?**  Additional question if the answer was not exhaustive:  Would you prefer financial or other incentives? |
| --- |

- - definition / general description: external strategies (e.g. rewarding, engaging) to influence behaviour via tangible or non-material methods.
  - example:

„Ultimately, that is the feasibility in the daily routine. […] for me it is always an incentive to feel more confident about what I am doing, to feel effective and not to miss out on something. That is enough for me.“

„Yes, if for example there was a better compensation over the compulsory insurance system.“

# Identifying guideline-related requirements of the PCP

- definition / general description of the cluster: includes all those aspects of PCPs attitudes, experiences, opinions or expectations which can be linked to the characteristics of the guideline. The aspects shall be structured according to the AGREE II. framework[[4](#_ENREF_4)] of guideline development. The description of the factors includes the AGREE II. general definition, and explanation for specific aspects the regarding the PCP`s scope.

### Referring Interview part

| *3. Question - knowledge and attitudes regarding the vertigo-guideline*  Perhaps you know that a S1 guideline of vertigo diagnosis and therapy is already available. This was created by the German Society of Neurology. An S3 guideline possibly better adapted to the needs of family practice is drafted currently by the German Society of General Medicine and Family Medicine. S3 guidelines are also based on a higher level of evidence than S1 guidelines. The publication of this guideline is planned for 2016.  Main question: What is your opinion with regard to these vertigo guidelines**?**  Supplementary questions if needed:   - What do you think about the content, the quality and reliability of currently available S1 dizziness guideline? - How do you rate their relevance in your practice? - What do you expect from the DEGAM guideline? |
| --- |

### Factors belonging to the cluster:

##### Domain 1. Scope and Purpose

- - definition / description: “The health question(s) covered by the guideline and the population (patients, public, etc.) to whom the guideline is meant to apply is specifically described. Item content includes the following criteria:
    - target population specified (e.g. clinical condition, severity/stage of disease, comorbidities, excluded populations)
    - health intent(s) (i.e., prevention, screening, diagnosis, treatment, etc.)
    - intervention(s) or exposure(s)
    - expected benefit or outcome
    - comparisons (if appropriate)
    - health care setting or context”

i.e. in our case the points 1-5 are self-explanatory, the point 6 would specify whether the guideline is relevant for and targeted to the primary health care; fitting to the opportunities/circumstances of the setting and to the competence/activity sphere of the PCPS

- - example:

„to get the guideline editors to the point of not drifting into scientific spheres, but stay grounded with both their feet“

„(DEGAM) they are always very practical“

##### Domain 2. Stakeholder Involvement

- - definition / description: “The guideline development group includes individuals from all the relevant professional groups, including target and patients; The views and preferences of the target population have been sought.” – i.e. in our case the PCP-involvement in guideline development has been/ should be ensured
  - example:

“ with those colleagues who actually do their job everyday in a general practice, not in a city with many organisational possibilities, but ideally on the countryside, and take their suggestions into account.““

„A distribution of the guideline to people from the field of general practice, asking them to check this guideline for practicability and to courageously and shamelessly criticize it.“

##### Domain 3. Rigour of Development

- - definition / description: “Systematic methods were used to search for evidence; The methods for formulating the recommendations are clearly described; There is an explicit link between the recommendations and the supporting evidence; The guideline has been externally reviewed by experts prior to its publication; A procedure for updating the guideline is provided.” – i.e. the level of evidence the guideline is based upon is high; and besides, the PCPs are also convinced in this regard.
  - examples

„exclusively the quality of a guideline“

„Then you start asking yourself, what is really evidence-based.“

##### Domain 4. Clarity of Presentation

- - definition / description: “The recommendations are specific and unambiguous; The different options for management of the condition are clearly presented; Key recommendations are easily identifiable” – i.e. the content (what to do, how to treat, which solution to choose depending on the patient’s condition etc.) is clear and understandable for the PCPs
  - example:

„Unambiguous, clear, straightforward and not further complicating indications for how I can establish a vertigo diagnosis out there in the general practice among three different patients.“

„Yes, to proceed in a standardised way“

##### Domain 5. Applicability

- - definition / description: “The guideline describes facilitators and barriers to its application. The guideline provides advice and/or tools on how the recommendations can be put into practice. The potential resource (cost) implications of applying the recommendations have been considered.” – i.e. it can easily be introduced in the primary care setting, thanks to “user-friendly” supportive tools, algorithms, summaries, check lists; describes practical patient paths, defines measurable monitoring criteria etc.
  - example:

„a structured diagnostic algorithm, graded according to evidence-based data, what are the most probable causes, what are the less probable causes.“

„And the quality of a guideline fits on one Din-A4 page.“

##### Domain 6. Editorial Independence

- - definition / description: “The views of the funding body have not influenced the content of the guideline.; Competing interests of guideline development group members have been recorded and addressed.” – i.e. the guideline is not influenced by financial interest and this is clear and transparent for the PCPs
  - example:

“in a very, very pharma-critical approach“

“ Just always have the expectation that this is not led by interests“

# Intervention opinions/preferences

Intervention methods were listed according to the Cochrane Effective Practice and Organisation of Care Group (EPOC)[[5](#_ENREF_5)] taxonomy of interventions. For characterising the methods, the review of Grimshaw[[6](#_ENREF_6)] provides details.

## Professional interventions

- definition / general description of the cluster: this question covers the PCPs attitudes and expectations regarding an intervention supporting the guideline implementation; with special attention to acceptance and applicability.

### Referring Interview part

| *7. Question - Preferences regarding the intervention*  Main question: **What form of guideline implementation would you prefer?**  If a decisive answer is provided, then ask:   - How would you characterise this method (i.e. how long, how many times, by whom and by what means, depending on what was mentioned)? - Is there any other additional method what you would find supportive and fits into your practice? - Do you think that the most acceptable form of implementation is in the same time also the most effective?   If no clear answer/preference is provided, then list the main methods of intervention:  I would now like to mention a few methods, please let me know which you prefer. Please interrupt me when I call a method that you find it helpful.   - Distribution of educational materials - Educational meetings (which form do you prefer, e.g. personal, e-learning...) - Local consensus processes in terms of content and of the implementation of the guideline - Educational outreach visits/ counselling by experts in the your practice - Involvement of Local opinion leaders - Patient mediated information - Audit and feedback - Reminders(electronically (e.g. Apps), by phone, in writing) - Measures to change existing attitudes and perspectives - Use of mass media to increase the awareness of the problem |
| --- |

### Factors belonging to the cluster:

#### Distribution of educational materials

- - definition / description: Distribution of published or printed recommendations for clinical care, including audiovisual materials and electronic publications. The materials may have been delivered personally or through mass mailings.
  - Factors that may influence resource use:
    - Format of educational materials (e.g. printed, audio-visual).
    - Method of distribution (e.g. by mail, personally delivered).
    - The number of copies of a given set of guidelines, the number of sets of guidelines and the number of times you are distributed.
  - example:

„good materials to be downloaded are what I need“

#### Educational meetings

- definition / description: Providers participating in conferences, lectures, workshops or traineeships, outside their practice setting.
- Factors that may influence resource use:
  - - Location, frequency and length of meetings. How much time would you dedicate….
    - Format of meetings (large didactic lecture, small interactive workshops).
    - webinar or personal?
    - credit point?
  - example:

„ rather personal advanced trainings, because it is possible to ask questions “

#### Local consensus processes

- - definition / description: Inclusion of participating providers in discussion to ensure that they agreed that the chosen clinical problem was important and the approach to managing the problem was appropriate.
  - example:

„„And if, in the context of guideline implementation, I bring in the colleagues from the respective specialties, to whom we refer or whom we consult now […] and if they have the opportunity to give feedback and work it in, then you can use your network in a positive way.”

#### Outreach visits

- definition / description: Use of a trained person who meets providers in their practice settings to give information with the intent of changing the provider’s practice. The information given may have included feedback on the performance of the provider(s). The meeting may be with an individual provider, or groups of providers.
- Factors that may influence resource use:
  - - Location, frequency and length of outreach visits (i.e. do they visit all PCPs in a practice at one meeting, do they visit consultants one by one?).
    - Number of outreach workers.
    - who are the visitors (credibility, acceptance)
  - example:

„a counselling hotline from an expert“

„the real vertigo experts won’t go into the practice““

#### Local opinion leaders

- - definition / description: Use of providers nominated by their colleagues as ‘educationally influential’. The investigators must have explicitly stated that their colleagues identified the opinion leaders.
  - Factors that may influence resource use:
    - Opinion leader (OL) activities (e.g. meetings, visits).
    - Number of opinion leaders.
    - who do they accept in this role?
  - example:

„ I am not very interested, in the claims of some self-proclaimed experts. So, what I want to say, if then, I have to comply with DEGAM recommendations, which means, general guidelines, but just any opinions, if that is an opinion leader or not, it is not really of interest to me.”

#### **Patient mediated interventions**

- - definition / description: New clinical information (not previously available) collected directly from patients and given to the provider e.g. depression scores from an instrument
  - example:

„ a structured questionnaire, that [...] would be helpful “

#### Audit and feedback

- definition / description: A summary of clinical performance over a specified period given to a provider. The summary may include recommendations for clinical action.
- The information may have been obtained from medical records, computerised databases or observations from patients. The feedback may include summaries of the clinical performance at the level of the individual provider, a group of providers, the practice, the institution or region. The recipient of the feedback may be the individual provider, a group of providers, the practice, the institution or region.
- Factors that may influence resource use:
  - - Frequency of the audit and feedback (balancing educational effect and getting tired from it).
    - Method of audit, related to data needed to measure the behaviour (e.g. manual audit of sample of medical records, use of routinely collected computerised data, PACT).
    - Format of feedback, produced by whom (printed report to individual/institution, meeting/briefing at individual level to institutional level).
  - example:

„that is always good” (ironically)“

#### Reminders

- - definition / description: Patient- or encounter-specific information provided verbally, on paper or on a computer screen, which is designed or intended to prompt a health professional to recall information. This would usually be encountered through their general education, in the medical records or through interactions with peers, and so remind them to perform or avoid some action to aid individual patient care.
  - Factors that may influence resource use:
    - Frequency and number of reminders/prompts.
    - Format and method of reminders/prompts (e.g. computer-generated printed checklist
    - attached to patient medical records by clerking staff, online prompt during patient encounter).
  - example:

„ Locally server based using the server software, that would be cool of course“

“ I am not a fan of it. That is what pharmaceutical companies like to do: Think of this and that“

#### Marketing

- definition / description: Use of personal interviewing, group discussion (‘focus groups’), or a survey of targeted providers to identify barriers to change and subsequent design of an intervention that addresses identified barriers.)
  - example:

„the more it is brought to the attention that there are guidelines and that they are specifically designed for primary care, the better“

#### Mass media

- - definition / description: (i)varied use of communication that reached great numbers of people including television, radio, newspapers, posters, leaflets, and booklets, alone or in conjunction with other interventions; (ii) targeted at the population level.
  - example:

“I do not believe in using mass media to inspire the wish for treatment. You know what mass media are good for? Prevention.“

## Other type of interventions

### Referring Interview part

| In addition to these professional interventions, there are also other approaches to support the implementation of guidelines. What methods would you prefer here? Please interrupt me again as soon as you feel a method as effective.   - Financial intervention - Organisational interventions in the primary care setting   - Provider orientated interventions   - Patient-orientated interventions   - Structural measures in the health care system - Regulatory interventions |
| --- |

### Factors belonging to the cluster:

### Financial interventions

- - definition / description: various means to create financial interest for complying with the required (i.e. in our case the guideline-adherent) behaviour, supporting either the service provider or the patient
  - example:

„if this additional effort is also compensated“

“What do you mean by financial interventions? To receive money? I refuse this [...] I cannot be bought“

### Organisational interventions:

- - definition / description: re-structuring services to improve care (e.g. revision of professional roles or substitution, staff reorganisation, case management including co-ordination of assessment, treatment and arrangement for referrals and follow-up; changes in physical structure, facilities and equipment; improving medical records systems (e.g. changing from paper to computerised records, patient tracking systems); but involving also consumer participation or dealing with patients’ suggestions and complaints)
  - example:

„A specialized walk-in clinic or structure, which is specialized only in vertigo in all its facets and eventually, if the patient enters through the front door, he leaves through the back door with some kind of finding.”

„You would need to re-organize our health care system, if we had less patient contacts, we could do more about it.“

### Regulatory interventions

- - definition / description: any intervention that aims to change health services delivery or costs by regulation or law (these interventions may overlap with organisational and financial interventions).
  - example:

„Yes. In any case. Because if I send a vertigo patient to the neurologist and he sends him to the radiologist and the radiologist sends him to the ear-nose-throat specialist, I'm very unhappy. So that is also something, what somehow should be prevented”

### Else (including refusal of any type of intervention)

*Remark: this category was added during the coding process.*

- - example:

“No. Just a guideline is needed which is understandable, applicable, uncomplicated, and therefore acceptable”

## Double label: indicating previous knowledge and attitude

##### spontaneously mentioned

##### stated as main preferred method

# References

1. Michie S, van Stralen MM, West R: **The behaviour change wheel: a new method for characterising and designing behaviour change interventions**. *Implementation science : IS* 2011, **6**:42.

2. Michie S, Johnston M, Abraham C, Lawton R, Parker D, Walker A, Psychological Theory G: **Making psychological theory useful for implementing evidence based practice: a consensus approach**. *Quality & safety in health care* 2005, **14**(1):26-33.

3. Damschroder LJ, Aron DC, Keith RE, Kirsh SR, Alexander JA, Lowery JC: **Fostering implementation of health services research findings into practice: a consolidated framework for advancing implementation science**. *Implementation science : IS* 2009, **4**:50.

4. Brouwers MC, Kho ME, Browman GP, Burgers JS, Cluzeau F, Feder G, Fervers B, Graham ID, Grimshaw J, Hanna SE *et al*: **AGREE II: advancing guideline development, reporting and evaluation in health care**. *CMAJ : Canadian Medical Association journal = journal de l'Association medicale canadienne* 2010, **182**(18):E839-842.

5. Effective Practice and Organisation of Care: **EPOC Taxonomy**. In*.*; 2015.

6. Grimshaw JM, Thomas RE, MacLennan G, Fraser C, Ramsay CR, Vale L, Whitty P, Eccles MP, Matowe L, Shirran L *et al*: **Effectiveness and efficiency of guideline dissemination and implementation strategies**. *Health technology assessment (Winchester, England)* 2004, **8**(6):iii-iv, 1-72.
