# Supplementary material for: Barriers and facilitators for the management of vertigo: a qualitative study with primary care providers
Source: Implement Sci. 2018 Feb 8;13:25. doi: 10.1186/s13012-018-0716-y (PMC5806383; doi:10.1186/s13012-018-0716-y)
Supplement: Supplementary file 3 — COM-B aspects of vertigo management in the primary care setting. This table lists content analysis results including English translations of exemplary citations for the COM-B aspects of vertigo management in the primary care setting. (DOCX 56 kb) [file 13012_2018_716_MOESM3_ESM.docx]

Additional files

Additional file 3. COM-B aspects of vertigo management in the primary care setting

| **Content analysis results including exemplary citations for COM-B aspects of vertigo management in the primary care setting** | |
| --- | --- |
|  | **English translation of original German citations** |
| **BEHAVIOUR (challenges in vertigo management)** | |
| 1. **Diagnostics** |  |
| lack of standardised procedure / algorithm | PCP4: „I see potential for improvement here, because I don’t have a standardised program. It is all still very intuitive when it comes to diagnostics.“ |
| Difficult cause identification due to unspecific symptom | PCP5: „That is sometimes quite difficult because […] it just is not fully clear what type of vertigo that is.“  PCP10: „Because there is no sense in curing the symptom if I don’t find the cause.“  PCP4: „The main problem is that we often don’t know exactly what we are talking about, if a patient says he is dizzy. […] The patient describes a whole range of sensations with that […] and even with targeted questions and investigations you are not really sure in the end, where does the phenomenon vertigo come from, and what does the patient mean by it?“ |
| identification of red flags | PCP9: „Ultimately, you have to exclude that some serious disease causes the vertigo. That is […] the danger with that is, that you might overlook something.“  PCP4: „having a little help […] where are the alarm signals, when do I have to react, what should I not miss, when can I rather wait and see.“ [Clarity of presentation] |
| 1. **Therapy** |  |
| patient groups: chronic vertigo / functional vertigo / vertigo in older patients | PCP2: „I don’t find it difficult at all.“  PCP3: „Difficult.“  PCP3: „You have to differentiate. For acute vertigo, it is not too difficult because the treatment regimen is relatively clear. For chronic vertigo it is difficult. And first of all also vertigo in older patients, that often is very resistant to treatment and that makes it difficult.“ |
| co-medication | PCP3: „If you actively ask what else they are taking, that is relevant […] which can also influence other medication [...]. You just have to keep an eye on what patients are taking without us knowing about it.“ |
| lack of treatment effectiveness | PCP9: „There are patients whom you just can’t treat.“ [Diagnostics] |
| 1. **Health care system** | |
| fragmented diagnostic process | PCP7: „The problem in vertigo management is, that with a referral, the vertigo is assigned to a certain specialty. And in case of doubt, the neurologist just notes that the vertigo is not related to his specialty. Period. The system leads to the fact that he does not think a step further: Where do we go from here? But instead he will just send the patient back. And the same goes for the ENT specialist, cardiologist, angiologist and so on. That means, that a crazy amount of time is lost through the recurrent returns of the patient to the PCP practice, just because we have a system which demands and promotes the narrowmindedness of each and every specialty. Say, I do my part, I check on my part, and if everything is fine there, I am done. And that, of course, is problematic for the patient.“  PCP9: „Difficult, as most of them [patients] really go through this specialist cycle.“  PCP7:„But the problem in part also lies in the time and resources, i.e. you will automatically refer the patient, although you could also do everything by yourself, just for time and structural reasons.“ [Diagnostics] |
| waiting time for specialists | PCP12: „I would just like to mention the specialist appointments […] because it takes very, very long to get an appointment and then it will be investigated and treated […] with disinterest, and the patients will ultimately come back without a result.“ |
| differing financial incentives | PCP7: „That in private health insurance it is easier to offer a service and get an individually calculated compensation per service, because it is possible to settle it as a single benefit, while in the compulsory insurance system the EBM (Einheitlicher Bewertungsmaßstab / Uniform Assessment Standard) provides a flat-rate system which is more difficult..So these are certainly factors [..] which I do not necessarily find positive, when an accounting system influences medical methodology. But I think, it does. I think if we don’t face this, we are blending out a part of reality.“ [Diagnostics] |
| availability of services in rural areas | PCP11: „We are on the countryside, more access to […] gymnastics groups, where you really do exercises and learn and have an opportunity for exchange, also in the sense of self-help. That would be good.“ |
| 1. **Patient** |  |
| Severity of symptoms | PCP3: „And to have an eye on how much the patient is affected by his symptoms. If it is an acute rotary vertigo, then the patient is so affected that they come here anyway and want to clear it up, and then is goes relatively fast.“ |
| Co-morbidities | PCP7: „If the patient is not very mobile, if the patient has other co-factors which make one suspected diagnosis far more probable than another, you will say, okay, then we do that here in our practice first and then we will see. So there are certainly factors which are encouraging.“ |
| patient focus on pharmacotherapy | PCP3: „Because I realised, overall, that the patients often have medical demands […]. And there seems to be quite a big market out there at the moment for some unspecific vertigo medications. That seems to be on the rise.“ [Therapy] |
| patient willingness to pay | PCP5: „and then the medications are partly only over-the-counter medications which means that the patients have to pay them themselves, and that is sometimes problematic.“ [Therapy] |
| Patient compliance | PCP5: „if certain diagnostic procedures are dictated in which the patients in the end are not ready to participate. Specifically patients are rather restrictive when it comes to extensive imaging diagnostics. They rather want a quick fix and do not want so much diagnostics.“ [Diagnostics][Scope and Purpose][Applicability][Social opportunity] |
|  | |
| **CAPABILITY** | |
| 1. **Psychological capability** | |
| awareness about guideline | PCP3: „To know that there is such a guideline is always the first step“ |
| handling competing priorities | PCP7: „Once I picture the practice, I see 50 or 100 things in front of me, where we would need guidelines with algorithms which should be implemented. […] I think vertigo has a good chance because it is a topic where many people find themselves in deep water […] but on the other hand, from a psychological perspective, you often tend to tackle the easy things. I mean, writing SOPs for bladder infections into your quality management system is far easier.“ |
| emotional prioritising | PCP7: „One barrier is certainly the overload with multiple problems and reasons for consultation, which are also perceived differently. […] Like palliative care, […] these topics have whole different emotional coverage, and you have to surpass these topics first.“ |
| psycological capacity / coping capacity | PCP7: „And that is difficult, because brainwise […] everyone is operating at full capacity already.“ [Physical opportunity] |
| Readiness to participate in continuous education | PCP3: „[Y]ou have to continue your education. You have to be up to date. And then you can also easily work with guidelines.“ |
| Self-discipline | PCP11: „In my perspective, guidelines are just a matter of discipline.“ |
| Organisation / general management skills | PCP3: „For me that depends on the PCP. And […] on the support of the practice team. And on being well organised.“ |
| 1. **Physical capability** |  |
| ability to perform tests | PCP8: „Sonography of the carotid artery, for example, that we cannot do, we are not trained for that.“  PCP1: „Or to perform the head impulse test. Testing something which you haven’t learned neurologically, I wouldn’t dare do that in the practice, and if I get into trouble that will cost me a lot of time.“  PCP7:„It is a difficult question, because eventually you could do an extensive part of the vertigo diagnostics also in the GP practice and it would make sense. […] But also part of the problem are time and resources.“ [Diagnostics] |
|  |  |
| **OPPORTUNITY** |  |
| 1. **Social Opportunity** |  |
| support of practice team | PCP3: „of course it also depends on the support of the practice team“ |
| exchange with colleagues | PCP11: „if you have a good quality circle, which works well on primary care topics, that is great, if you are able to involve them.“ |
| cooperation of the patients | PCP12: „if it [is] comprehensible for the patient“  PCP7: „a positive factor […] is just the physician-patient relationship, if the motivation or the treatment contract and the personal relationship is positive and close and good.“ |
| the PCP's specialist network | PCP7: „And if, in the context of guideline implementation, I bring in the colleagues from the respective specialties, to whom we refer or whom we consult now […] and if they have the opportunity to give feedback and work it in, then you can use your network in a positive way. But if your network blocks you and says: „Listen, do what you want in your PCP practice, we will do it differently anyways.“, then it is a problem, and of course, then it is a barrier.“ [Local consensus processes] |
| DEGAM | PCP7: „I am a PCP, and I am a member of the German College of General Practitioners and Family [DEGAM] Physicians and as such, if there is a DEGAM guideline which I would like to implement, then I will just do it.“ |
| independence | PCP11: „Actually, as a single-handed practice it is really me who is doing this. And I decide it and I do the training of my employees.“ [Psychological capability] |
| 1. **Physical opportunity** |  |
| time – work balance | PCP7: „The most important barrier is time. Because, if a vertigo patient sits in front of you, usually 3,4,5,6,7,8,9,10 more patients with many other problems are waiting outside, such that you just don’t have the time and peace to dedicate yourself to the topic as much as the topic deserves it. That is one very decisive barrier.“  PCP4: „Barrier is simply the work load caused by the sheer number of patients to be cared for[…], so, many things stay left behind and you can’t deal with everything in such a structured way as you would like to.“  PCP4: „Less patients. […] Germany is absolutely leading in Europe regarding the number of patient contacts per day. With a lower frequency we could also engage better with every single person in every single case.“ |
| electronic availability of knowledge resources | PCP3: „[…] just the fact that I have internet access and am able to have a look, because this […] is still not the case in many PCP practices […]. Because if they don’t work online, then they also don’t have the information about such a guideline. Personally, I often just go online and read up on things.“ |
| staff | PCP7: „For this project of integrating a vertigo guideline in this practice here, I could probably employ an assistant or a part-time resident […] for about half a year and they would still not be bored. […] For one guideline I would probably need an additional part-time position for half a year.“  PCP8: „We have a relatively large team, also relatively many assistants […] If […] investigations could be delegated, then it would indeed be possible.“ |
| practice facilities | PCP9: „It depends what the guideline prescribes and demands. I mean, with regard to equipment or examinations, depending on if that is feasible and can be implemented in my practice.“ [Applicability] |
| specialised (tertiary) health care availability | PCP7: „A specialised walk-in clinic or structure, which is specialised only in vertigo in all its facets and […] if the patient comes in on one side, he leaves from the other side with some kind of insight.“ [Organisational interventions] |
| financing | PCP10: „[…] And the benefactors don’t cooperate in these cases anymore. You have to acknowledge that as well. The financial resources are also somewhat limited.“ |
|  |  |
| **MOTIVATION** |  |
| intrinsic motivation without specific argumentation / own free decision / ("true" automatic motivation) | PCP7: „And for the rest, it is just endogenous motivation, just to say, we want that, we want that.“  PCP10: „The possibilities? That question is asked in the wrong direction. Please, that is a question of willingness.“  PCP10: „Well, and I don’t think that guidelines should substitute thinking.“ [Psychological capability]  PCP7: „Of course, if I look back after the implementation, and say: „look at this, we have made it“, that is great, then you are happy, that motivates you, but the inner temptation beforehand is huge.“ |
| increasing self-confidence, self-assurance | PCP4: „for me it is always an incentive to feel more confident about what I am doing, to feel effective and not to miss out on something. That is enough for me.“ [Incentives] |
| increased knowledge | PCP6: „of course I stay informed in my daily routine, you don’t want to stand still.“ |
| success in treatment / possibility of providing better care | PCP12: „if in therapy there would also be successes“ |
| automation /simplifying practice | PCP7: „If guidelines were implemented in the practice management software, that is always great, […] then the only thing you need to find the right button to press, and if you have done this often enough, you will know.“ [Psychological capability] |
| protection in case of legal responsibility | PCP6: „and […] to be able, in case of need, to document that I have acted in accordance with the guideline.“ [Incentives] |
| prevalence of disease, contribution to the caseload | PCP11: „Well, one motivator is that it is so frequent“ |
| financing | PCP8: „[…] if this additional effort is also compensated.“ [Incentives] |
| credibility | PCP7: „Well-ordered workflows, everybody does the same thing, everyone does it in a similar way, the patients go to two different doctors and each of them tells them the same thing. The patients arrive back at the reception counter and the assistants also say the same thing. You know, it is also an increase in quality perceived from outside, if all are on the same page.“ [Social opportunity] |
| constructive criticism | PCP7: „We will not be able to do this by finger pointing. You cannot say: „Dear PCPs, you are bad here and here and there, and here and there you are not doing well, and here and there you are missing a good work flow and here and there you only work with your gut-feeling and not evidence“, if we do that, everyone blocks, rightly so, and they will say: „Just leave me alone. I work sixty, seventy hours a week and you come by and tell me, how to do it right. Why don’t you do it yourself?“ [Physical opportunity] |
|  |  |
|  | |
| **INCENTIVES** | |
| applicability of the guideline / simplifying practice | PCP2: „exclusively the quality of a guideline. And the quality of a guideline fits on one DIN-A4 page.“ [Rigour of Development][ Applicability]  PCP1: „feasiblity, that the guideline really relies on experience and has been tested accordingly and shows success as well.“ |
| instructions on guideline implementation | PCP8: „It would definitely be an incentive to have it all explained, how to implement it all, […] specifically targeted to the PCP practice“ [Educational meetings] |
| Success in treatment | PCP9: „If it is something reasonable, a reasonable source of instruments to eventually treat my patients better. Then there are no ifs, ands or buts.“ [Applicability][Motivation] |
| financial incentive | PCP5: „Yes, if for example there was a better compensation over the compulsory insurance system.“  PCP10: „No, I tell you how it is. I cannot be bought.“ |
| remote support | PCP12: „If, e.g. I just had a contact person.“ [Organisational interventions]  PCP12: „Yes, a counseling hotline from an expert.“ [Organisational interventions][Educational outreach visits] |
