# Supplementary material for: Barriers and facilitators for the management of vertigo: a qualitative study with primary care providers
Source: Implement Sci. 2018 Feb 8;13:25. doi: 10.1186/s13012-018-0716-y (PMC5806383; doi:10.1186/s13012-018-0716-y)
Supplement: Supplementary file 4 — PCPs’ guideline expectations in vertigo management. This table lists English translations of characteristic citations for the PCPs’ view about guideline-related facilitators and barriers grouped according to the AGREE II. Framework. (DOCX 47 kb) [file 13012_2018_716_MOESM4_ESM.docx]

Additional files

Additional file 4. PCPs` guideline expectations in vertigo management

| **Aspects** (grouped according to AGREE II. Framework) | **Characteristic citations for the PCPs` view about the guideline-related facilitators and barriers [code co-occurrence, reflecting the connections between constructs depicted in the model, Fig. 1]** |
| --- | --- |
|  | **English translation of the German citations** |
| Domain 1: Scope and Purpose | |
| - - adjustment of the guidelines to the reality/circumstances of primary care | PCP4: „DEGAM…they are always very practical.“  PCP2: „to get the guideline editors to the point of not drifting into scientific spheres, but stay grounded with both their feet.“ |
| Domain 2: Stakeholder Involvement | |
| - - editors’ insufficient focus on the PCPs` needs and circumstances | PCP2: „Because one gets the impression that the colleagues who create the guidelines often don’t work in everyday practice anymore, under the pressures and impressions of doing the practical job.“ |
| - - involvement of PCPs in guideline development including those from settings with lower resources | PCP2: „A distribution of the guideline to people from the field of general practice, asking them to check this guideline for practicability and to courageously and shamelessly criticise it.“  PCP2: „with those colleagues who actually do their job everyday in a general practice, not in a city with many organisational possibilities, but ideally on the countryside, and take their suggestions into account.“ [Physical opportunity] |
| Domain 3: Rigour of Development | |
| - - perceived evidence-base of the guideline | PCP2: „exclusively the quality of a guideline“  PCP10: „Then you start asking yourself, what is really evidence-based.“ |
| Domain 4: Clarity of presentation | |
| - - style of presenting the information | PCP2: „Unambiguous, clear, straightforward and not further complicating indications for how I can establish a vertigo diagnosis out there in the general practice among three different patients.“  PCP4: „And also having a little bit of help, a thing that these DEGAM guidelines are achieving, is, where are the alarm signals, when do I have to react, what must I not miss, when could I also just wait and see.“ [Diagnostics]  PCP5: „Yes, to proceed in a standardised way and that this could result in positive aspects for the patient, that therapy is optimised, that therapy gets more standardised.“ [Motivation]  PCP1: „the opinion or guideline which is most comprehensible to me“ [Psychological capability] |
| - - consideration of specific patient sub-groups | PCP1: „I don’t know to what extent experiences with older and very old patients are integrated in these guidelines“  PCP11: „Also with regard to these hybrid forms, aged persons and so on, what can I do in these cases? That is missing at the moment.“ |
| Domain 5: Applicability | |
| - - length of a guideline | PCP4: „if you have to read through a 300-page guideline by yourself and extract something for yourself, that is always difficult and slowing you down. Thus, if you have short forms, if you have ready-made instruments to which you can revert, then obviously that is helpful.“ [Psychological capability] |
| - - algorithm | PCP7: „a structured diagnostic algorithm, graded according to evidence-based data, what are the most probable causes, what are the less probable causes.“  PCP4: „And if, for example, they include good flow charts, when do I do what and how, in order to be able to integrate this in a standardised way.“ [Psychological capability]  PCP5: „if, again, certain diagnostic procedures are uniformly required, to which the patients eventually do not agree to participate. Especially patients are rather restrictive when it comes to extensive imaging diagnostics.“ [Patient challenges] |
| - - 1-page summary | PCP2: „And the quality of a guideline fits on one Din-A4 page.“ [Incentive] |
| - - patient information and self-help material | PCP10: „for the patients I wouldn’t openly offer any, because that generates the demand for treatment.“  PCP11: „And, yes, I think, or, I hope, that there will be new findings on what else you could do or advise. Activating self-help, such things, that would be important to me.“ [Therapy] |
| - - including information for / involvement of practice assistants | PCP7: „physician assistants. Because most workflow aspects are in their hands. I sit here and talk and maybe I do some diagnostics, but everything after that: „Which laboratory? Where do I go? Which referral? Whom do we call? When does he return? What to do with the results?“ and so on, that is a lot in the hands of the physician assistants. You shouldn’t underestimate that, actually they always need their own support for really implementing this in their everyday work.“ |
| - - Fitting to everyday practice | PCP5: „I would call it positive to have look at guidelines and to implement the corresponding things which fit into your practice.“ [Motivation]  PCP4: „Ultimately, that is the feasibility in the daily routine. You know, a guideline must be easily usable. It cannot be extremely complicated or laborious, because then you can’t integrate it in your daily routine […] that gets difficult for the complex problems like vertigo.“ [Incentive]  PCP4: „The most important thing is actually always the guideline: How practicable is it, how does it work under the absolutely limited resources which we have in practice, how well can it be implemented? I fit is extremely comprehensive, but then you need an hour per patient until you are done, then you can’t do it.“ [Phys. opportunity] |
| Domain 6: Editorial Independence | |
| - - from other specialities | PCP4: „that a specialist’s guideline is not implementable in general practice“  PCP7: „The S1 guideline is one out of one hundred recommendations for action and guidelines from all kinds of specialties, for which I as a PCP do not have the time or feel like dealing with it intensively and seriously, first of all because they have been compiled by a specialist.“ |
| - - from pharma industry | PCP7: „[DEGAM]…in a very, very pharma-critical approach.“  PCP10: „And then these guidelines often also lead to discussions which are not lead openly, because there are some financial interests in the background, and that includes interests of the pharmaceutical companies.“ |
