# Supplementary material for: Barriers and facilitators for the management of vertigo: a qualitative study with primary care providers
Source: Implement Sci. 2018 Feb 8;13:25. doi: 10.1186/s13012-018-0716-y (PMC5806383; doi:10.1186/s13012-018-0716-y)
Supplement: Supplementary file 5 — PCPs’ opinion about intervention methods. This table lists English translations of characteristic citations for the PCPs’ view on various intervention methods grouped according to the EPOC list of interventions. (DOCX 49 kb) [file 13012_2018_716_MOESM5_ESM.docx]

Additional files

Additional file 5. PCPs` opinion about intervention methods

|  | **Characteristic citations for the PCPs` view on various intervention methods [code co-occurrence reflects the connections between constructs depicted in the model, Fig. 1]** |
| --- | --- |
|  | **English translation of the German original citation** |
| 1. Professional interventions | |
| 1.1 Distribution of educational materials   - indicated as preferred method: 2 meaning units - favourable statements: 4 meaning units - negative statements: 3 meaning units | PCP4: „good materials to be downloaded are what I need“  PCP5: „emails“  PCP7: „I believe that for many practices it is too early for online methods and apps and the whole external data exchange and the interconnectedness does not yet work sufficiently well. But, true, that is something today where I might say in five years’ time that it is perhaps something good“ |
| 1.2 educational meeting   - indicated as preferred method: 4 meaning units - favourable statements: 10 meaning units - negative statements: 4 meaning units | PCP1: „rather personal advanced trainings, because it is possible to ask questions“  PCP3: „Totally depends on the age of the doctors […] is 62 now, […] couldn’t do anything with e-learning.“  PCP4: „even more advanced trainings, going somewhere, that becomes difficult“  PCP7: „some vague advanced trainings where you sit down and then you leave being as clever as before, that will get you nowhere“  PCP12: “a short training about vertigo diagnostics. And where you can look up things online, which tests I am supposed to do and how to do them best. By a specialist. Right, that would be important..“ [Psychological Capability]  PCP5: „If advanced trainings were offered through the local medical chapter and if one would go through the guideline also in a circle with colleagues, for example in a quality circle, and could integrate the colleagues‘ opinions.“ [Social opportunity]  PCP8: „Yes, well, if there was an advanced training, I would totally like it. That would definitely be an incentive, when someone explains to you how to implement that. And also precisely for PCPs, not for neurologists or something like that, but really what PCPs can do in the case of vertigo. I believe that would also help to implement things in practice.“ [Incentive] |
| 1.3 Local consensus process   - indicated as preferred method: 1 meaning units - favourable statements: 5 meaning units - negative statements: 4 meaning unit | PCP2: „to check this guideline for practicability and to courageously and shamelessly criticise it.“ “  PCP7: „And if, in the context of guideline implementation, I bring in colleagues from the respective specialties, to whom we refer or whom we consult now […] and if they have the opportunity to give feedback and work it in, then you can use your network in a positive way.“ [Social opportunity] |
| 1.4 Outreach visits   - indicated as preferred method: 0 meaning units - favourable statements: 5 meaning units - negative statements: 4 meaning units | PCP12: „a counselling hotline from an expert“ [Incentive]  „if I just, for example, have a contact person“ [Incentive]  PCP1: „the real vertigo experts won’t go into the practice“ |
| 1.5 local opinion leaders   - indicated as preferred method: 0 meaning units - favourable statements: 2 meaning units - negative statements: 6 meaning units | PCP6: „Since we don’t accept any pharmaceutical representatives in our practice anymore, that means, I am not very interested, in the claims of some self-proclaimed experts. So, what I want to say, if then, I have to comply with DEGAM recommendations, which means, general guidelines, but just any opinions, if that is an opinion leader or not, it is not really of interest to me.” [Social opportunity] |
| 1.6 Patient-mediated interventions   - indicated as preferred method: 0 meaning units - favourable statements: 4 meaning units - negative statements: 2 meaning units | PCP1: „a structured questionnaire, that [...] would be helpful“  PCP7: „So maybe the patient would have to get such a referral slip with which he goes from doctor to doctor where he says then: "Thank you, that you have examined me, look this all the diagnostic procedures I have undergone at the PCP, how should we proceed?” [Health care] |
| 1.7 Audit and Feedback   - indicated as preferred method: 0 meaning units - favourable statements: 3 meaning units - negative statements: 3 meaning unit | PCP3: “especially for a vertigo guideline I do not see a special need for this.”  PCP8: “that is always good” (ironically) |
| 1.8 Reminders   - indicated as preferred method: 0 meaning units - favourable statements: 6 meaning units - negative statements: 3 meaning units | PCP7: “Locally server based using the server software, that would be cool of course“  PCP10: “I am not a fan of it. That is what pharmaceutical companies like to do: ”Think of this and that”  PCP11: “and such an impulse you usually need. To take a look: What is really new? And if it is, for example, published in ZFA [Zeitschrift für Allgemeinmedizin / Journal for general medicine], that is just always great, you know.“ [Physical oppurtunity] |
| 1.9 Marketing   - indicated as preferred method: 0 meaning units - favourable statements: 6 meaning units - negative statements: 3 meaning units | PCP11: „the more it is brought to attention, that there are guidelines and that they are specifically designed for primary care, the better“  PCP10: “People do not even know what is wrong with them, and eventually they are provided with suggestions about what might be wrong with them” |
| 1.10 Mass Media   - indicated as preferred method: 0 meaning units - favourable statements: 1 meaning units - negative statements: 7 meaning units | PCP3 “Yes, actually with vertigo I would rather not do that, because it is such an unspecific symptom. If people’s attention is drawn to it even more, then… everybody is dizzy at some time. And if the awareness for this problem is raised, then people try to find a reason for something that would go away anyways, and then you have over-diagnostics. Thus, no.”  PCP10: “I do not believe in using mass media to inspire the wish for treatment. You know what mass media are good for? Prevention.” |
| 2. financial interventions   - indicated as preferred method: 1 meaning units - favourable statements: 6 meaning units - negative statements: 4 meaning units | PCP5: „If, for example, there is a better compensation over the compulsory health insurance system.“ [Incentive]  PCP7: „in my opinion, the external support in organisation, counselling and implementation is also a financial incentive, because I don’t have to pay for it.“ [Incentive]  PCP11: „I believe, that this will just lead to misuse. And it also doesn’t really change the mindset “ |
| 3. Organisational interventions   - indicated as preferred method: 1 meaning units - favourable statements: 10 meaning units - negative statements: 1 meaning units | PCP7: „the most effective would be an additional permanent position, financed by a public sponsor for implementing all guidelines in practice“  PCP10: “If you want to treat in a practice [...]according to guidelines, then you would actually need a computer program where you can enter the symptoms.” [Diagnostics/Therapy]  PCP7: „A specialised walk-in clinic or structure, which is specialised only in vertigo in all its facets and eventually, if the patient enters through the front door, he leaves through the back door with some kind of finding.” [Health care]  PCP4: „You would need to re-organise our health care system, if we had less patient contacts, we could do more about it.” [Physical opportunity]  PCP12: “If I could check with someone over the telephone or via email, what is actually meant by this and that, or that I am not of the same opinion, or if I am not coming to terms with a patient or if something is not clear.” [Incentive]  PCP1: „That would be possible, but it is hard to implement because of the practices’ heterogeneity.” |
| 4. Regulatory interventions   - indicated as preferred method: 0 meaning units - favourable statements: 4 meaning units - negative statements: 4 meaning units | PCP3: „No, I have no ideas regarding this.“  PCP12: „In general, that also makes sense“ |
